# TOX4 facilitates promoter-proximal pausing and C-terminal domain dephosphorylation of RNA polymerase II in human cells

**DOI:** 10.1038/s42003-022-03214-1

**Published:** 2022-04-01

**Authors:** Ziling Liu, Aiwei Wu, Zhen Wu, Talang Wang, Yixuan Pan, Bing Li, Xumin Zhang, Ming Yu

**Affiliations:** 1 Sheng Yushou Center of Cell Biology and Immunology, School of Life Sciences and Biotechnology, Shanghai Jiao Tong University, 200240 Shanghai, China; 2 State key Laboratory of Genetic Engineering, School of Life Sciences, Fudan University, 200438 Shanghai, China; 3 Department of Biochemistry and Molecular Cell Biology, Shanghai Jiao Tong University School of Medicine, 200025 Shanghai, China; 4 Ministry of Education Key Laboratory of Systems Biomedicine, Shanghai Jiao Tong University, 200240 Shanghai, China

**Keywords:** Transcription, Gene regulation

## Abstract

TOX4 is one of the regulatory factors of PP1 phosphatases with poorly understood functions. Here we show that chromatin occupancy pattern of TOX4 resembles that of RNA polymerase II (Pol II), and its loss increases cellular level of C-terminal domain (CTD) phosphorylated Pol II but mainly decreases Pol II occupancy on promoters. In addition, elongation rate analyses by 4sUDRB-seq suggest that TOX4 restricts pause release and early elongation but promotes late elongation. Moreover, TT-seq analyses indicate that TOX4 loss mainly decreases transcriptional output. Mechanistically, TOX4 may restrict pause release through facilitating CTD serine 2 and DSIF dephosphorylation, and promote Pol II recycling and reinitiation through facilitating CTD serines 2 and 5 dephosphorylation. Furthermore, among the PP1 phosphatases, TOX4 preferentially binds PP1α and is capable of facilitating Pol II CTD dephosphorylation in vitro. These results lay the foundation for a better understanding of the role of TOX4 in transcriptional regulation.

## Abstract (web summary)

As a role of TOX4, one of the regulatory proteins of PP1 phosphatases, in transcriptional regulation, authors here show that TOX4 restricts pause release and early productive elongation, and promotes Pol II recycling and transcriptional reinitiation.

## Introduction

Transcription can be divided into three stages, initiation, elongation, and termination^1^. Post-translational modifications of proteins, in particular phosphorylation, are known to play critical roles in transcription^2^. Pol II consists of 12 subunits, i.e., RPB1–12, and among them, RPB1 is the largest subunit that contains a catalytic domain and a C-terminal domain (CTD)^3^. The CTD contains a heptad peptide (Y^1^-S^2^-P^3^-T^4^-S^5^-P^6^-S^7^) that is repeated 26 and 52 times in budding yeast and human, respectively. The CTD plays critical and yet incompletely understood roles in gene expression and can be delicately regulated by dynamic phosphorylation of residues within this domain, most notably serine 2 (Ser-2) and serine 5 (Ser-5)^4,5^.

In the initiation of Pol II transcription, six general transcription factors (GTFs), i.e., TFIIA, TFIID, TFIIB, TFIIE, TFIIF, and TFIIH, form a pre-initiation complex (PIC) with Pol II for the recognition of a transcriptional start site (TSS) and the creation and stabilization of a transcription bubble^6^. In this process, CDK7 kinase, one of the subunits of TFIIH, plays a critical role by phosphorylating CTD Ser-5 to aid in promoter escape by Pol II^4^. In metazoans, elongation by Pol II includes three steps, i.e., promoter clearance^7^, promoter-proximal pause release and productive elongation^8^. Initiation and pause release are recognized as critical checkpoints of transcriptional regulation^8^. Binding of NELF and DSIF to elongation-competent Pol II 20–80 nt downstream of TSSs stabilizes its promoter-proximal pause, and the release requires kinase P-TEFb, a heterodimer of CDK9 kinase and Cyclin T1, and the PAF1 complex (PAF1C)^9^. P-TEFb phosphorylates NELF to promote its displacement from Pol II, the C-terminal repeat (CTR) of the SPT5 subunit of DSIF to convert DSIF from a negative elongation factor to a positive one, and Ser-2 of Pol II CTD, which is critical for the regulation of transcriptional elongation, RNA processing and transcriptional termination^8,10^. In contrast to their phosphorylation, dephosphorylation of DSIF, NELF, and CTD Ser-2 by phosphatases is less well understood. The PP2A-Integrator complex was found to regulate multistep of transcription by dephosphorylating the Pol II CTD and the SPT5 CTR^11–13^, and PP4 regulates pause release by dephosphorylating SPT5 Threonine 806 (Thr-806)^14^. In the termination of Pol II transcription, the PNUTS-protein phosphatase 1 (PP1) complex decelerates elongation by dephosphorylating SPT5 CTR, the CPSF complex cleaves pre-mRNA at a polyadenylation site, P-TEFb phosphorylates 5′-3′ exonuclease XRN2 to stimulate its activity, and XRN2 displaces Pol II from DNA by degrading Pol II-associated RNA^15–17^. Subsequently, Pol II CTD has to be dephosphorylated by phosphatases, including FCP1 and SCPs, to allow efficient Pol II reincorporation into PICs, but the underlying mechanism is incompletely understood^18^. Moreover, Ssu72, a phosphatase for CTD serines 5 and 7, enhances transcription directionality through facilitating promoter-terminator loop formation in yeast^19,20^.

Protein kinases and phosphatases mainly catalyze phosphorylation and dephosphorylation of serine (Ser), threonine (Thr), and tyrosine (Tyr) residues of proteins^21^. The substrate specificity of kinases and tyrosine phosphatases is determined by structural features of the enzymes themselves and regulatory proteins, but substrate specificity of Ser/Thr phosphatases is mainly dependent on regulatory proteins, which play targeting, substate specifying and inhibitory roles^22–24^. The PP1 family of Ser/Thr phosphatases contains PP1 α, β, and γ, which are ~90% identical in protein sequences, and is estimated to be responsible for the dephosphorylation of around 50% of the human phosphoproteome^23^. They were recently found to be able to form protein phosphatase 1 complex (PP1C), which contains one of the phosphatases and three regulatory proteins, PNUTS, TOX4, and WDR82^25^. Among the three regulatory proteins, WDR82 and PNUTS have been found to restrain transcription of enhancer RNA (eRNA) and promoter upstream transcript (PROMPT) by enforcing early termination and facilitate termination of mRNAs in murine macrophages^26^, PNUTS plays an important role in transcription and RNA processing partially by facilitating or restricting dephosphorylation by PP1 phosphatases^16,27^, PNUTS and WDR82 were recently shown to prevent transcription–replication conflicts by promoting Pol II degradation^28^, whereas the role of TOX4 in gene regulation is unknown.

To understand the role of TOX4 in gene regulation, we performed functional genomic and biochemical studies, and discovered that TOX4 may restrict pause release and early productive elongation through facilitating CTD Ser-2 and SPT5 Thr-806 dephosphorylation, and promote Pol II recycling and transcriptional reinitiation by facilitating CTD serines 2 and 5 dephosphorylation.

## Results

### Chromatin occupancy pattern of TOX4 resembles that of Pol II

**Fig. 1:**
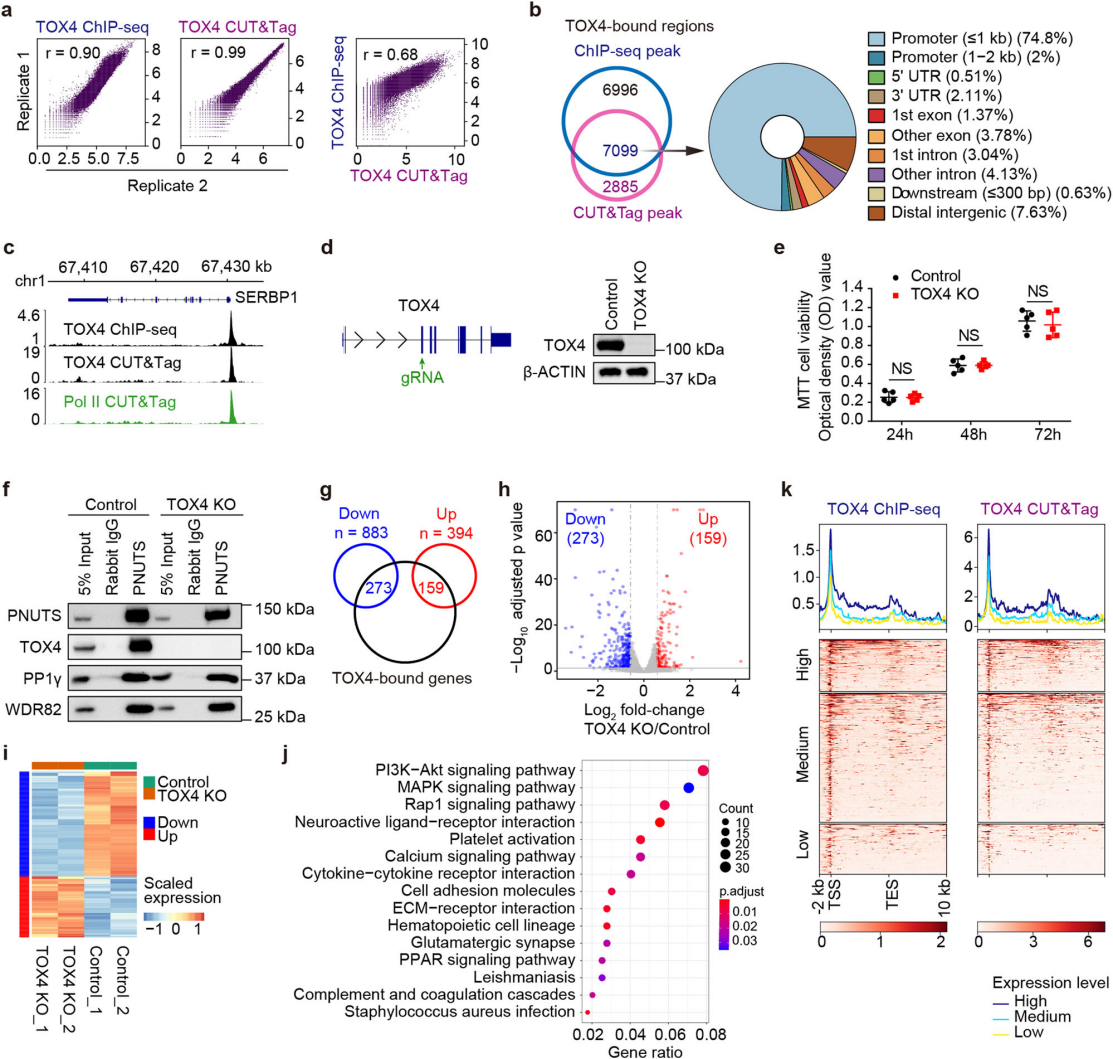
Chromatin occupancy pattern of TOX4 resembles that of Pol II. **a** Correlation plots for biological replicates of ChIP-seq (left) and CUT&Tag (middle) of TOX4, and for ChIP-seq versus CUT&Tag of TOX4 (right). **b** Annotation of TOX4 occupancy. Left: a Venn diagram showing overlap between TOX4 peaks identified by ChIP-seq and CUT&Tag, respectively, Right: TOX4 peak distribution across genomic features. **c** Normalized read distribution of TOX4 ChIP-seq (top), TOX4 CUT&Tag (middle) and Pol II CUT&Tag (bottom) within the *SERBP1* locus in K562 cells. **d** Generation and characterization of a TOX4 knockout K562 cell line. Left: Schematic of TOX4 knockout strategy. Right: Characterization of the TOX4 KO K562 cell line by Western blot. **e** Comparison of proliferation of control and TOX4 KO cells by MTT assays. Statistical significance was determined with a two-sided Student’s *t*-test; the centers and the error bars represent the mean and the SD of five independent experiments, respectively. NS: *P* ≥ 0.05. **f** Comparison of association of WDR82 or PP1γ with PNUTS by co-IP in control and TOX4 KO cells. **g** A Venn diagram showing overlaps between TOX4-bound genes and downregulated or upregulated genes of RNA-seq. **h** A volcano plot showing expression changes of TOX4-bound genes upon TOX4 loss. **i** A heatmap comparing expression of TOX4 direct targets in TOX4 KO versus control cells. **j** Pathway analysis results of downregulated genes upon TOX4 loss. **k** Meta-gene profiles and heatmaps of ChIP-seq (left) and CUT&Tag (right) each showing a positive correlation between TOX4 occupancies and mRNA levels of TOX4 direct targets in K562 cells. Genes were sorted according to TOX4 occupancy level detected by ChIP-seq (left) and CUT&Tag (right), respectively, in control cells. High: top 25% of the non-silent genes (mean TPM ≥ 1); Medium: non-silent genes between top 25% and bottom 25%; Low: bottom 25% of the non-silent genes or genes with mean TPM < 1.

The human erythroleukemic cell line, K562, which is widely used in functional genomic studies, was chosen to investigate the role of TOX4 in gene regulation. We started by analyzing chromatin occupancy of TOX4 by ChIP-seq and CUT&Tag. Correlation analyses of related biological replicates indicate that the data are highly reproducible, and correlation analysis of TOX4 ChIP-seq and CUT&Tag indicates that the data are reliable (Fig. 1a). Analyses of high confidence occupancy regions, defined as those detectable by ChIP-seq and CUT&Tag with FDR-adjusted *P* value < 10^−6^ and top 1% signal, respectively (Fig. 1b), discovered that it occupies most of the active genes from transcription start sites (TSSs) to several kilobases downstream of transcription end sites (TESs) (Fig. 1b, c, k), a pattern resembling that of Pol II, suggesting a close connection of TOX4 to Pol II transcription^9^. To facilitate subsequent functional studies, we generated a TOX4 knockout (KO) cell line using the CRISPR-Cas9 methodology (Fig. 1d). By MTT assays, we found that proliferation of K562 cells was minimally affected by TOX4 loss (Fig. 1e). To assess the effect of TOX4 loss on integrity of the PP1C, we performed co-IP experiments using an antibody against PNUTS in control and TOX4 KO cells. We found that interactions of WDR82 and PP1γ with PNUTS were unaffected in the absence of TOX4, suggesting that TOX4 loss does not affect the formation of the WDR82-PNUTS-PP1 complex (Fig. 1f).

To assess the effect of TOX4 loss on the transcriptome of K562 cells, we performed RNA-seq experiments. The numbers of upregulated and downregulated genes were 394 and 883, respectively, upon TOX4 loss with fold change ≥ 1.5 and FDR-adjusted *P* value < 0.05 (Supplementary Fig. 1a, b). Among them, the numbers of upregulated and downregulated TOX4 direct targets, defined as TOX4-bound genes with significant mRNA level changes upon its loss, were 159 and 273, respectively (Fig. 1g–i). In addition, pathway analysis of genes with significant expression changes revealed enrichment of genes in several signaling pathways, notably the PI3K-AKT pathway (Fig. 1j). The association of PP1C with Pol II^29^ and the positive correlation between Pol II occupancy and mRNA level of genes^30^ led us to investigate if there is any correlation between TOX4 occupancy and mRNA level of genes. A positive correlation was uncovered by comparative analyses of occupancy data of ChIP-seq or CUT&Tag and expression data of RNA-seq (Fig. 1k).

### TOX4 may facilitate Pol II CTD dephosphorylation

**Fig. 2:**
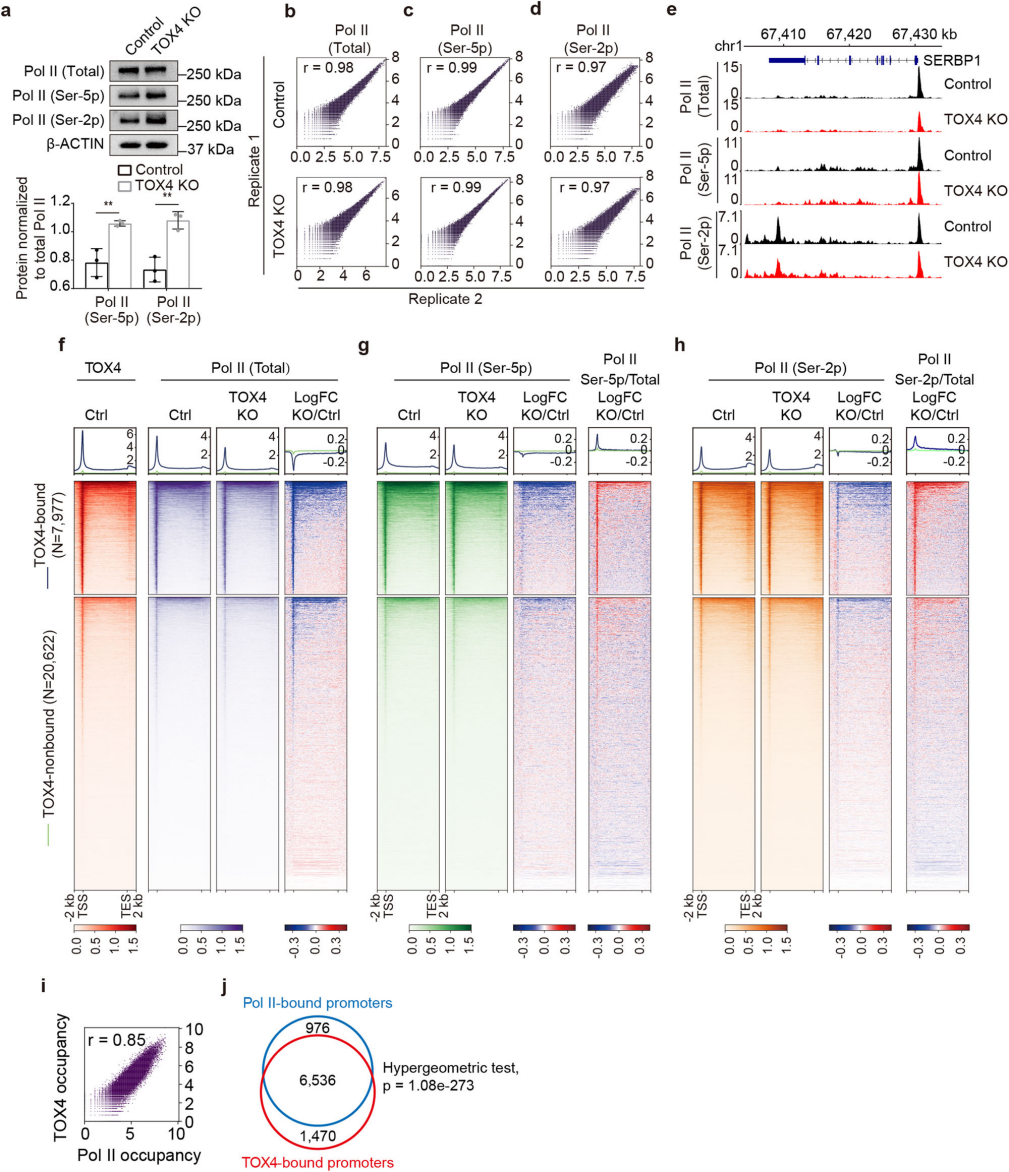
TOX4 may facilitate Pol II CTD dephosphorylation. **a** Comparison of cellular levels of total, Ser-5 phosphorylated and Ser-2 phosphorylated Pol II by Western blot in control and TOX4 KO cells. Top: Western blot images, Bottom: A bar graph showing relative levels of Ser-5 phosphorylated Pol II and Ser-2 phosphorylated Pol II quantified by ImageJ in control and TOX4 KO cells. Pictures are representative of three independent experiments. Statistical significance was determined with a two-sided Student’s *t*-test; the centers and the error bars represent the mean and the SD, respectively. *P < 0.05, **P < 0.01. **b**–**d** Correlation plots for biological replicates of CUT&Tag of total (**b**), Ser-5 phosphorylated (**c**), and Ser-2 phosphorylated (**d**) Pol II. **e** Normalized read distribution of CUT&Tag of total, Ser-5 phosphorylated and Ser-2 phosphorylated Pol II within the *SERBP1* locus in TOX4 KO versus control cells. **f**–**h** Genome-wide meta-gene profiles and heatmaps of CUT&Tag comparing chromatin occupancies of total (**f**), Ser-5 phosphorylated (**g**), and Ser-2 phosphorylated (**h**) Pol II in TOX4 KO versus control (Ctrl) cells. A genome-wide meta-gene profile and a heatmap of TOX4 CUT&Tag in control cells are placed to the left of those of Pol II to facilitate comparison of TOX4 and Pol II occupancies. Genes were sorted by total Pol II CUT&Tag signal in control cells. **i** A correlation plot of CUT&Tag data of TOX4 and Pol II. **j** A Venn diagram showing overlap between TOX4-bound and Pol II-bound promoters. Statistical significance of the overlap between them was determined with a Hypergeometric test.

The identification of TOX4 as one of the regulatory subunits of PP1C^25^, the suggested role of PP1C in Pol II CTD dephosphorylation^31^, and the resemblance between TOX4 and Pol II occupancies (Fig. 1c, k) led us to assess whether TOX4 loss affects Pol II CTD phosphorylation. By Western blot, we found that TOX4 KO increased cellular levels of Ser-5 phosphorylated Pol II and Ser-2 phosphorylated Pol II while level of total Pol II was unaffected (Fig. 2a), suggesting that TOX4 may facilitate CTD dephosphorylation by PP1 phosphatases. Considering that elongating Pol II is highly phosphorylated on Ser-2 compared to initiating Pol II, the increase of Ser-2 phosphorylation also suggests that TOX4 restricts elongation. However, it is also known that chromatin occupancy changes of proteins are not always consistent with their cellular level changes. To examine the effect of TOX4 loss on Pol II occupancy, we performed CUT&Tag experiments for total, Ser-5 phosphorylated and Ser-2 phosphorylated Pol II with high reproducibility (Fig. 2b–d). We found that TOX4 loss markedly reduced total Pol II occupancies on most of the TOX4-bound genes (Fig. 2e, f). TOX4 loss also slightly reduced occupancies of Ser-5 phosphorylated Pol II and Ser-2 phosphorylated Pol II on a subset of TOX4-bound genes (Fig. 2e, g, h), which although are inconsistent with the Western blot results (Fig. 2a), suggest that they are consequences of the greater reduction of total Pol II occupancy. Subsequent normalization of occupancies of Ser-2 phosphorylated Pol II and Ser-5 phosphorylated Pol II individually to total Pol II occupancy discovered increased relative occupancies of them (Fig. 2g, h), supporting the above-mentioned idea. Moreover, the CUT&Tag results of total, Ser-5 phosphorylated and Ser-2 phosphorylated Pol II were validated by ChIP-qPCR on a number of genes (Supplementary Fig. 2a–c). Furthermore, comparative analysis of CUT&Tag data of TOX4 and total Pol II discovered high degree of correlation between them (Fig. 2i), and ~87% Pol II-occupied promoters are co-occupied by TOX4, further suggesting a close connection of TOX4 to Pol II transcription (Fig. 2j).

To determine if the effects of TOX4 loss on the cellular level and the chromatin occupancy of Pol II are bona fide (not the off-target effects of the gRNA) and to analyze the effects of acute TOX4 depletion on transcription, we tried to generate a cell line with acute inducible TOX4 degradation on the basis of the auxin-inducible degron (AID) 2 system^32^. Unfortunately, the resulting K562-AID-TOX4 cell line has leaky degradation problem (Supplementary Fig. 3a). Nevertheless, it can be used to determine whether the results obtained from the TOX4 KO cell line are off-target effects of the gRNA or not.

By Western blot, we found that TOX4 downregulation increased cellular levels of Ser-5 phosphorylated Pol II and Ser-2 phosphorylated Pol II while level of total Pol II was unaffected (Supplementary Fig. 3a), which are consistent with the results of TOX4 KO. By CUT&Tag, we found that TOX4 downregulation decreased total Pol II occupancy although to a lesser degree compared with that of TOX4 KO (Supplementary Fig. 3b, e, f). Moreover, occupancies of Ser-5 phosphorylated Pol II and Ser-2 phosphorylated Pol II were minimally affected (Supplementary Fig. 3c–e, g, h). However, normalization of occupancies of Ser-5 phosphorylated Pol II and Ser-2 phosphorylated Pol II individually to that of total Pol II discovered increased relative occupancies of them (Supplementary Fig. 3g, h), which are also consistent with the results from control and TOX4 KO cells. Together, these data suggest that our findings in TOX4 KO cells are bona fide and not the off-target effects of the gRNA.

### TOX4 may promote promoter-proximal pausing of Pol II

**Fig. 3:**
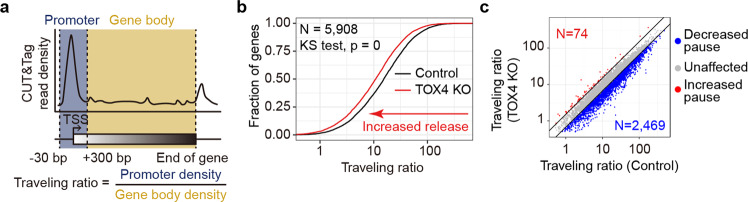
TOX4 facilitates promoter-proximal pause of Pol II. **a** Schematic representation describing the calculation of traveling ratio (TR) at each Pol II-bound gene. **b** A cumulative distribution plot comparing Pol II TRs of TOX4-bound genes in control and TOX4 KO cells. **c** A scatter plot comparing Pol II TRs of TOX4-bound genes in TOX4 KO versus control cells.

**Fig. 4:**
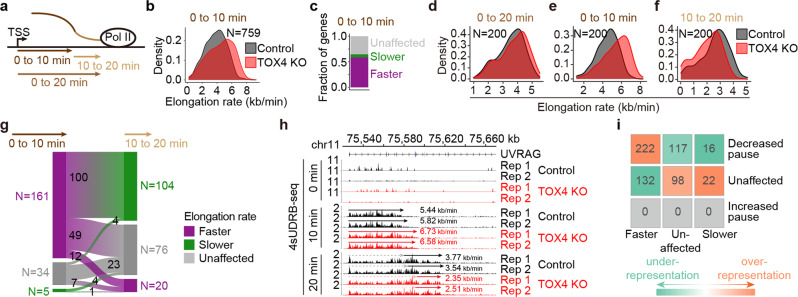
TOX4 may restrict early elongation but promote late productive elongation of Pol II. **a** Schematic representation of experimental design of the 4sUDRB-seq. Labeled RNA was extracted at 0, 10, and 20 min after DRB removal. **b** Density plots comparing Pol II elongation rates of 759 TOX4-bound genes in control and TOX4 KO cells, for which high confidence elongation rates from 0 to 10 min after DRB removal could be determined. **c** A bar graph categorizing Pol II elongation rate changes of the above-mentioned 759 TOX4-bound genes in TOX4 KO versus control cells. **d**–**f** Density plots comparing Pol II elongation rates from 0 to 20 min (**d**), 0 to 10 min (**e**), and 10 to 20 min (**f**) after DRB removal of 200 TOX4-bound genes in control and TOX4 KO cells, for which high confidence elongation rates from 0 to 10 min and 0 to 20 min could be determined. **g** A Sankey diagram visualizing Pol II elongation rate changes from 0 to 10 min and 10 to 20 min of 200 TOX4-bound genes described in **d**–**f**. Heights of rectangles and widths of “streams” between rectangles each are proportional to gene counts, which are shown either aside of the rectangles or inside of the “streams”. **h** Normalized 4sUDRB-seq read distribution at 0, 10 and 20 min after DRB removal within the *UVRAG* locus in control and TOX4 KO cells. **i** A heatmap of standardized residuals of the chi-square test of independence between Pol II elongation rate changes from 0 to 10 min after DRB removal and Pol II TR changes upon TOX4 loss. Overlapping gene counts between categories are shown in the cells.

Decreased total Pol II occupancy (Fig. 2e, f), increased cellular level (Fig. 2a) and relative chromatin occupancy of CTD Ser-2 phosphorylated Pol II (Fig. 2e, h), and established connections between PNUTS-PP1 complex and transcriptional elongation/termination^16,26^ led us to further investigate the role of TOX4 in elongation. To evaluate if TOX4 affects pause release, we calculated traveling ratios (TRs) of Pol II^33^ for TOX4-bound genes using the total Pol II CUT&Tag data (Fig. 3a). TOX4 loss markedly decreases Pol II TRs of 41.8% of the TOX4-bound genes (Fig. 3b, c), indicating a role in restricting pause release. However, besides increase of pause release, decrease of transcriptional initiation and/or initiation to elongation transition would also decrease Pol II TRs. In addition, decrease of initiation would decrease Pol II occupancy on TSSs and gene bodies, whereas increase of pause release usually decreases Pol II occupancy on TSSs while increases Pol II occupancy on gene bodies. Therefore, genes with decreased Pol II TRs and increased Pol II occupancy on gene bodies are more likely to have increased pause release upon TOX4 loss (Further discussed in Figs. 4 and 7).

### TOX4 may restrict early elongation but promote late productive elongation

Since pause release and productive elongation are regulated by reversible phosphorylation of several common amino acid residues, including CTD Ser-2 and SPT5 Thr-806^14^, If TOX4 restricts pause release, there is a high probability that it also restricts productive elongation. To assess the effects of TOX4 loss on productive elongation, we performed 4sUDRB-seq experiments^34^ with three time points, 0, 10, and 20 min (Fig. 4a). Considering that elongation rates are ~3.8 kb/min in human cells^35^, we therefore only considered genes longer than 30 kb for analyzing elongation rates from 0 to 10 min and genes longer than 60 kb for analyzing elongation rates from 0 to 20 min. We found that TOX4 loss increased elongation rates of TOX4-bound genes from 0 to10 min (Fig. 4b), and among the 759 nonoverlapping genes with lengths over 30 kb and showing clear front borders of elongation, 58% exhibited increased rates in contrast to 5.8% exhibited decreased rates (Fig. 4c), not only suggesting a role of TOX4 in restricting productive elongation but also supporting the findings that TOX4 may restrict pause release. Additionally, we found that although TOX4 loss increased overall rates from 0 to 20 min (Fig. 4d), the rates actually increased from 0 to 10 min but decreased from 10 to 20 min (Fig. 4e, f), suggesting that TOX4 promotes late productive elongation (elongation on gene body regions dozens of kilobases downstream of TSSs) besides restricting early elongation (pause release plus early productive elongation). Specifically, upon TOX4 loss, among the 200 nonoverlapping TOX4-bound genes with lengths over 60 kb and showing clear front borders of elongation, the numbers of genes with decreased, unaffected and increased rate were 5, 34, and 161, respectively, from 0 to 10 min, and were 104, 76, and 20, respectively, from 10 to 20 min (Fig. 4g). Moreover, among the 76 genes with unaffected rate from 10 to 20 min, 49 of them actually exhibited increased rate from 0 to 10 min, indicating rate decreases after the initial rate increases. The *UVRAG* gene is a typical example of those genes with increased rate from 0 to 10 min and decreased rate afterwards (Fig. 4h).

To evaluate the probability that TOX4 restricts pause release (using TR as the readout) and early productive elongation (using elongation rate as the readout) of the same gene, we performed chi-square test of independency between changes of TR and elongation rate of Pol II. We found a significant association between decreased TRs and increased elongation rates (chi-square test, *P* = 0.016) (Fig. 4i), suggesting that TOX4 is likely to restrict pause release and productive elongation of the same gene.

### TOX4 may restrict transcriptional elongation by facilitating DSIF dephosphorylation

DSIF and NELF phosphorylation is required for pause release besides that of Pol II CTD Ser-2^8,36^, and DSIF phosphorylation has also been found to stimulate productive elongation^16^. Therefore, TOX4 may also restrict pause release by facilitating DSIF and NELF dephosphorylation and the subsequent productive elongation by facilitating DSIF dephosphorylation. A high-quality antibody against phosphorylated Thr-806 of SPT5, one of the known targets of PP1 phosphatases and a site that has been characterized in pause release and productive elongation, is available^14^, so we analyzed if TOX4 regulates SPT5 Thr-806 dephosphorylation.

**Fig. 5:**
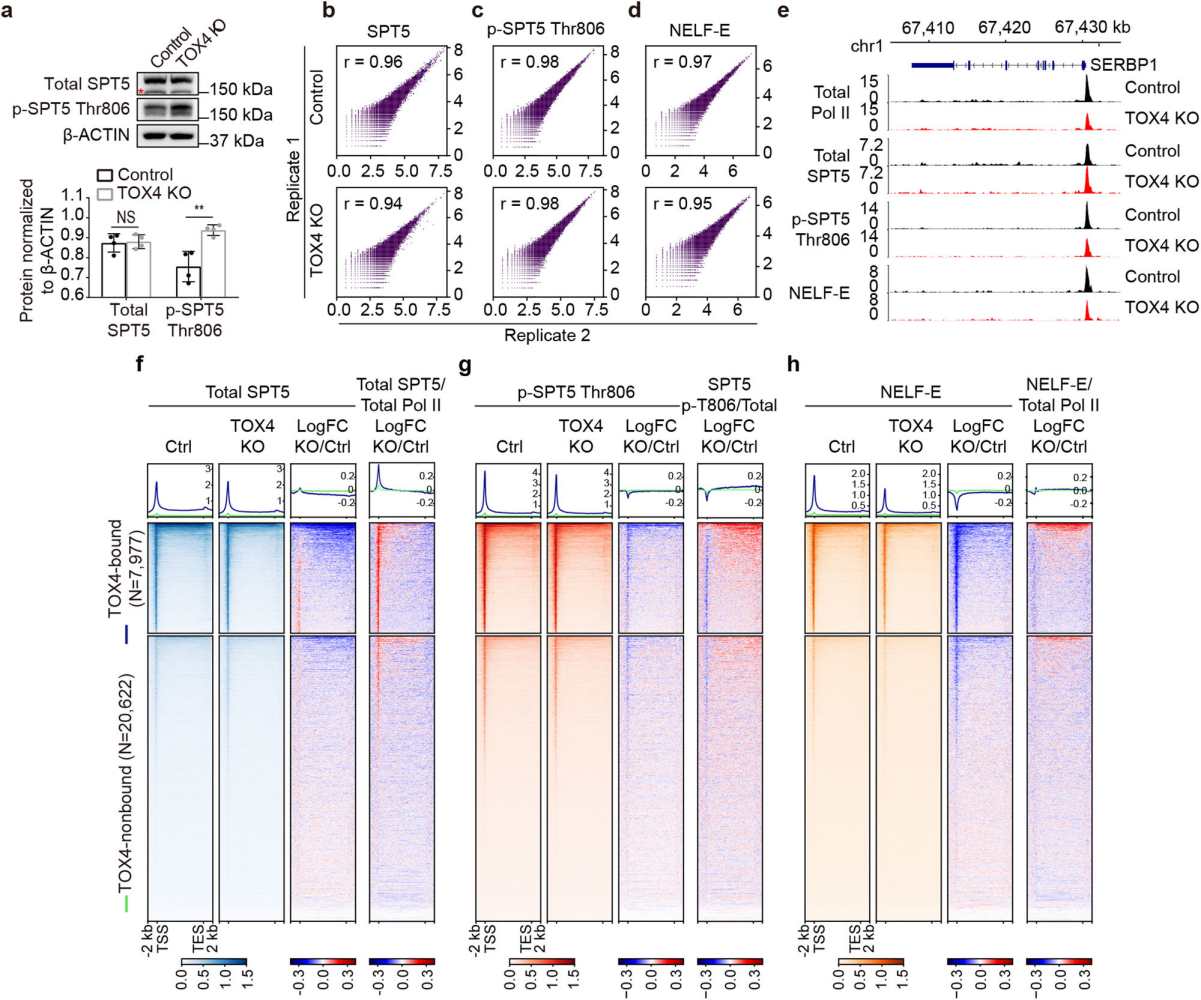
TOX4 may restrict early elongation by facilitating SPT5 Thr-806 dephosphorylation. **a** Comparison of cellular levels of SPT5 and p-SPT5 Thr-806 by Western blot in control and TOX4 KO cells. Top: Western blot images with nonspecific bands highlighted by a “*”, Bottom: A bar graph showing relative levels of p-SPT5 Thr-806 quantified by ImageJ in control and TOX4 KO cells. Pictures are representative of four independent experiments. Statistical significance was determined with a two-sided Student’s *t*-test; the centers and the error bars represent the mean and the SD, respectively. NS: *P* ≥ 0.05, *P < 0.05, **P < 0.01. **b**–**d** Correlation plots for biological replicates of CUT&Tag of SPT5 (**b**), p-SPT5 Thr-806 (**c**), and NELF-E (**d**). **e** Normalized CUT&Tag read distribution of Pol II, SPT5, p-SPT5 Thr806 and NELF-E within the *SERBP1* locus in TOX4 KO versus control cells. **f**–**h** Genome-wide meta-gene profiles and heatmaps of CUT&Tag comparing chromatin occupancies of SPT5 (**f**), p-SPT5 Thr-806 (**g**), and NELF-E (**h**) in TOX4 KO versus control cells. Genes were sorted by total Pol II CUT&Tag signal in control cells.

We first examined effects of TOX4 loss on cellular levels of SPT5 and p-SPT5 Thr-806 by Western blot, and found no effect on SPT5 level, but slightly increased p-SPT5 Thr-806 level (Fig. 5a). However, chromatin occupancy changes of proteins are not always consistent with their cellular level changes. To determine the effects of TOX4 loss on occupancies of SPT5 and p-SPT5 Thr-806, we subsequently performed CUT&Tag experiments with high reproducibility (Fig. 5b, c). We found that TOX4 loss slightly increased SPT5 occupancy near TSSs, reduced SPT5 occupancy downstream of TSSs (Fig. 5e, f), slightly decreased p-SPT5 Thr-806 occupancy near TSSs, and minimally affected p-SPT5 Thr-806 occupancy downstream of TSSs (Fig. 5e, g), suggesting an increase of p-SPT5 Thr-806 occupancy relative to SPT5 occupancy on gene bodies. This increase was confirmed by normalization of p-SPT5 Thr-806 occupancy to that of SPT5 (Fig. 5g). Together, these results suggest that TOX4 restricts pause release and early productive elongation of Pol II partially by facilitating SPT5 Thr-806 dephosphorylation. Nevertheless, considering that effects of TOX4 loss on cellular level (Fig. 5a) and relative occupancy of p-SPT5 Thr-806 (Fig. 5g) are small, these effects may also be indirect and the consequences of increased pause release.

DSIF and NELF occupancies are mainly dependent on Pol II occupancy^37^, so decreased total Pol II occupancies on genes (Fig. 2e, f) would decrease SPT5 occupancies. However, SPT5 occupancies on TSSs actually slightly increased upon TOX4 loss and the increases are much greater when normalized to total Pol II occupancies (Fig. 5e, f), suggesting that TOX4 restricts SPT5 recruitment, but the mechanisms are unknown. To determine if TOX4 regulates NELF occupancy, we performed CUT&Tag experiments for NELF-E, one of the subunits of NELF (Fig. 5d). We found that TOX4 loss markedly reduced NELF-E occupancy (Fig. 5e, h), but relative NELF-E occupancy exhibited little change (Fig. 5h), suggesting that TOX4 does not regulate NELF recruitment.

### TOX4 may not promote late elongation by decompacting chromatin

Our 4sUDRB-seq analyses suggest that TOX4 promotes late elongation (Fig. 4f–h), but the underlying mechanism is unknown. Considering that HMG box-containing proteins are capable of decompacting chromatin^38^ and that Tox1 and 2 have been shown to regulate chromatin accessibility^39–42^, we therefore hypothesized that TOX4 promotes late productive elongation by decompacting chromatin. To test this idea, we performed ATAC-seq experiments with high reproducibility (Supplementary Fig. 4a). A set of consensus peaks were obtained by merging peaks from control and TOX4 KO cells for the identification of regions with accessibility change (Supplementary Fig. 4b). We found that TOX4 loss affected neither global accessibility nor distribution of accessible sites across genomic features (Supplementary Fig. 4c, d). With fold change ≥ 2 and FDR-adjusted *P* value < 0.01, the numbers of sites with decreased and increased accessibility were 6830 and 4882, respectively (Supplementary Fig. 4e). In addition, comparative analyses of sites with accessibility change and sites with TOX4 occupancy identified 1172 and 131 TOX4-bound accessible sites with decreased and increased accessibility, respectively (Supplementary Fig. 4f, g), suggesting that TOX4 mainly decompacts chromatin. Notably, comparison of genomic distribution of less accessible, unaffected and more accessible sites with TOX4 occupancy uncovered that a high percentage of sites on gene bodies (from 2 kb downstream of TSSs to 300 bp downstream of TESs) became less accessible upon TOX4 loss (Supplementary Fig. 4h), suggesting that TOX4 may be responsible for decompacting chromatin downstream of TSSs.

To test this idea, we generated metagene profiles with the ATAC-seq data on an average gene (from 2 kb upstream of TSS to 10 kb downstream of TES), an average promoter (2.5 kb upstream and 2 kb downstream of TSS) and an average gene without TSS (from 2 kb downstream of TSS to 5 kb downstream of TES), respectively. TOX4 KO slightly increased accessibility near TSSs, but markedly decreased accessibility of gene bodies and regions several kilobases downstream of TESs of a subset of TOX4-bound genes (Supplementary Fig. 4i–k), supporting the idea that TOX4 may decompact chromatin downstream of TSSs. However, examination of the 200 nonoverlapping TOX4-bound genes with lengths over 60 kb and showing clear front borders of elongation for gene body accessibility changes identified more genes with decreased late elongation rate and increased body accessibility than genes with decreased late elongation rate and body accessibility (Supplementary Fig. 4l), suggesting that TOX4 does not promote late elongation through increasing accessibility downstream of TSSs. Specifically, among the 153 genes with decreased late productive elongation rate (104 genes with increased or unaffected elongation rate from 0 to 10 min and decreased elongation rate from 10 to 20 min plus 49 genes with increased elongation rate from 0 to 10 min and unaffected elongation rate from 10 to 20 min), the numbers of genes with increased, unaffected, and decreased accessibility were 31, 115, and 7, respectively; among the 47 genes with unaffected or increased late productive elongation rate, the numbers of genes with increased, unaffected, and decreased accessibility were 11, 33, and 3, respectively (Supplementary Fig. 4l). Moreover, comparative analyses of TOX4 CUT&Tag, ATAC-seq, and RNA-seq data identified 69 direct targets with accessibility change (Supplementary Fig. 4m), which is much smaller than the total number of direct target genes, suggesting that TOX4 mainly regulates transcription independent of its accessibility modulating capability. Furthermore, there is an expected positive correlation between changes of chromatin accessibility and mRNA level of TOX4 direct targets (Supplementary Fig. 4m).

### TOX4 preferentially binds PP1α and promotes Pol II CTD dephosphorylation

Although TOX4 loss increased not only cellular levels (Fig. 2a) but also relative occupancies of Ser-5 phosphorylated Pol II and Ser-2 phosphorylated Pol II (Fig. 2g, h), the effect of TOX4 loss on chromatin occupancy of PP1 phosphatases is undetermined. To this end, we performed CUT&Tag experiments for PP1α, β, and γ with high reproducibility (Supplementary Fig. 5a–c). We found that 69, 31, and 91% of the genes with TOX4 occupancy are co-occupied by PP1α, β, and γ, respectively. The reason why lower percentage of TOX4 peaks are co-occupied by PP1α or β is that antibodies of PP1α and β are not as good as PP1γ antibody for CUT&Tag. Conversely, 81% of PP1α-bound, 92% of PP1β-bound and 76% of PP1 γ-bound genes are co-occupied by TOX4 (Supplementary Fig. 5d–f). Together, these data suggest high-frequency of co-occupancy between TOX4 and any of the PP1 phosphatases.

**Fig. 6:**
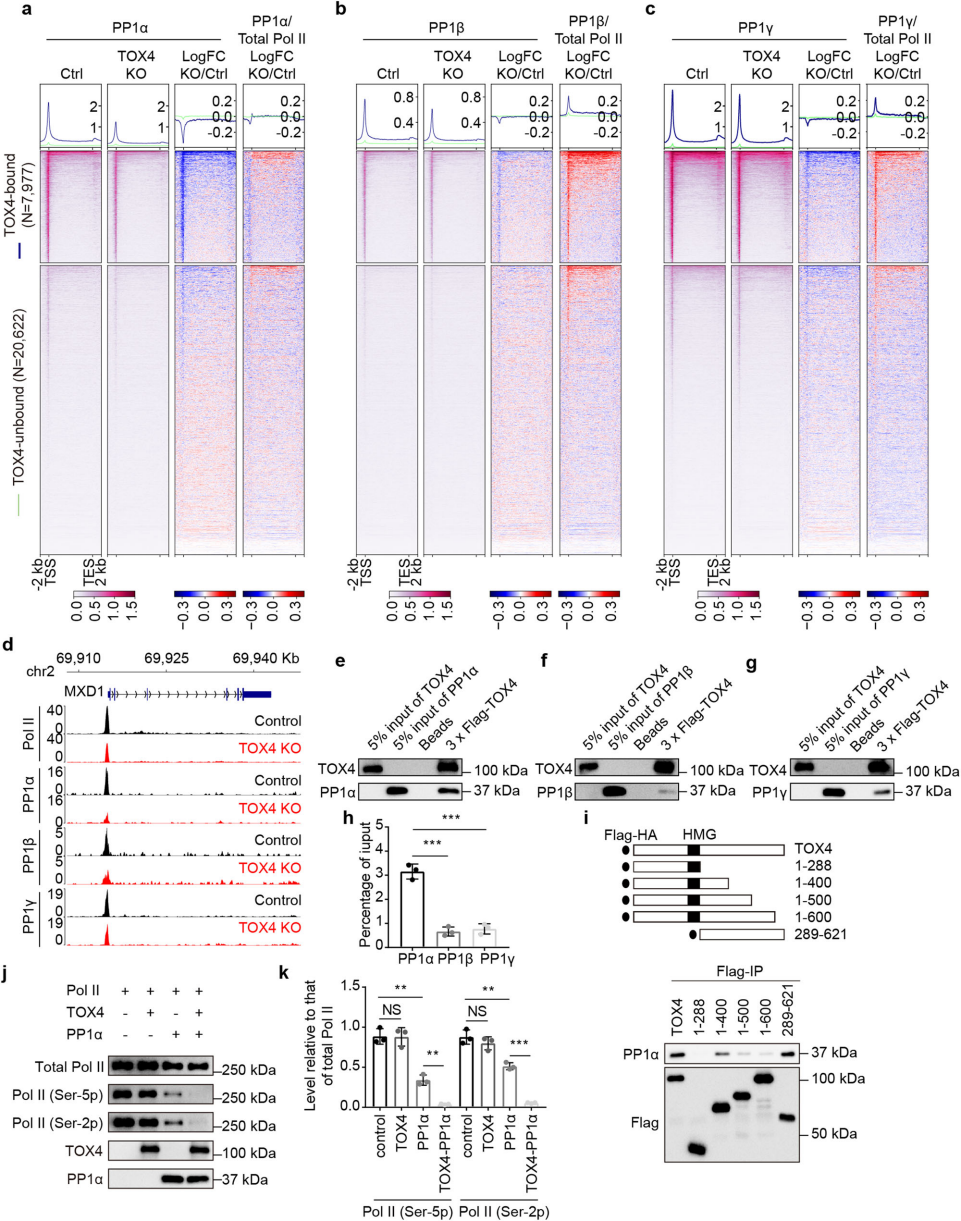
TOX4 preferentially binds PP1α and facilitates Pol II CTD dephosphorylation. **a**–**c** Genome-wide meta-gene profiles and heatmaps of CUT&Tag comparing chromatin occupancies of PP1α (**a**), β (**b**), and γ (**c**) and relative chromatin occupancies of them to that of total Pol II in TOX4 KO versus control cells. Genes were sorted by total Pol II CUT&Tag signal in control cells. **d** Normalized CUT&Tag read distribution of Pol II, PP1α, PP1β, and PP1γ within the *MXD1* locus in TOX4 KO versus control cells. **e**–**g** Western blot analyses of in vitro binding assays between TOX4 and PP1α (**e**), β (**f**), or γ (**g**). **h** A bar graph comparing percentage of PP1α, β, or γ co-immunoprecipitated with TOX4 in in vitro binding assays quantified by ImageJ. **i** Western blot analyses of co-IP experiments between PP1α and fragments of TOX4. **j** Western blot analyses of in vitro phosphatase assay using purified PP1α, TOX4, TOX4-PP1α, and Pol II. **k** A bar graph comparing levels of Ser-2 phosphorylated Pol II and Ser-5 phosphorylated Pol II in in vitro phosphatase assays quantified by ImageJ. Pictures in **e**–**g**, and **j** are representative of three independent experiments. Statistical significance in **h**, **k** was determined with a two-sided Student’s *t*-test; the centers and the error bars represent the mean and the SD, respectively. NS: *P* ≥ 0.05, *P < 0.05, **P < 0.01, ***P < 0.001.

Importantly, we found that TOX4 loss markedly decreased PP1α occupancy while decreased occupancies of PP1β and γ to a lesser degree (Fig. 6a–d), suggesting that TOX4 preferentially regulates PP1α recruitment and that among the PP1 phosphatases, TOX4 preferentially binds PP1α. To test this idea, we first individually purified TOX4, PP1α, PP1β, and PP1γ from High Five cells infected with the corresponding baculovirus, and subsequently performed in vitro binding assays. We found that although TOX4 is capable of binding any of the PP1 phosphatases, its binding to PP1α is much stronger than that to PP1β or γ (Fig. 6e–h), supporting the idea that TOX4 preferentially binds PP1α. To determine which region of TOX4 is responsible for interaction with PP1α, we constructed a series of TOX4 mutants and performed co-IP experiments using them individually with PP1α, and found that the C-terminal region (amino acids 601–621) is responsible for the interaction (Fig. 6i). To determine how TOX4 affects PP1α activity, we performed in vitro phosphatase assay using PP1α, TOX4, and TOX4–PP1α complex purified from baculoviruses infected High Five cells, respectively, and Pol II purified from 293T cells, and found that TOX4 facilitates CTD Ser-2 and Ser-5 dephosphorylation (Fig. 6j, k). Together, these results suggest that TOX4 preferentially binds PP1α and promotes Pol II CTD dephosphorylation.

### TOX4 may promote Pol II recycling and transcriptional reinitiation through facilitating CTD dephosphorylation

**Fig. 7:**
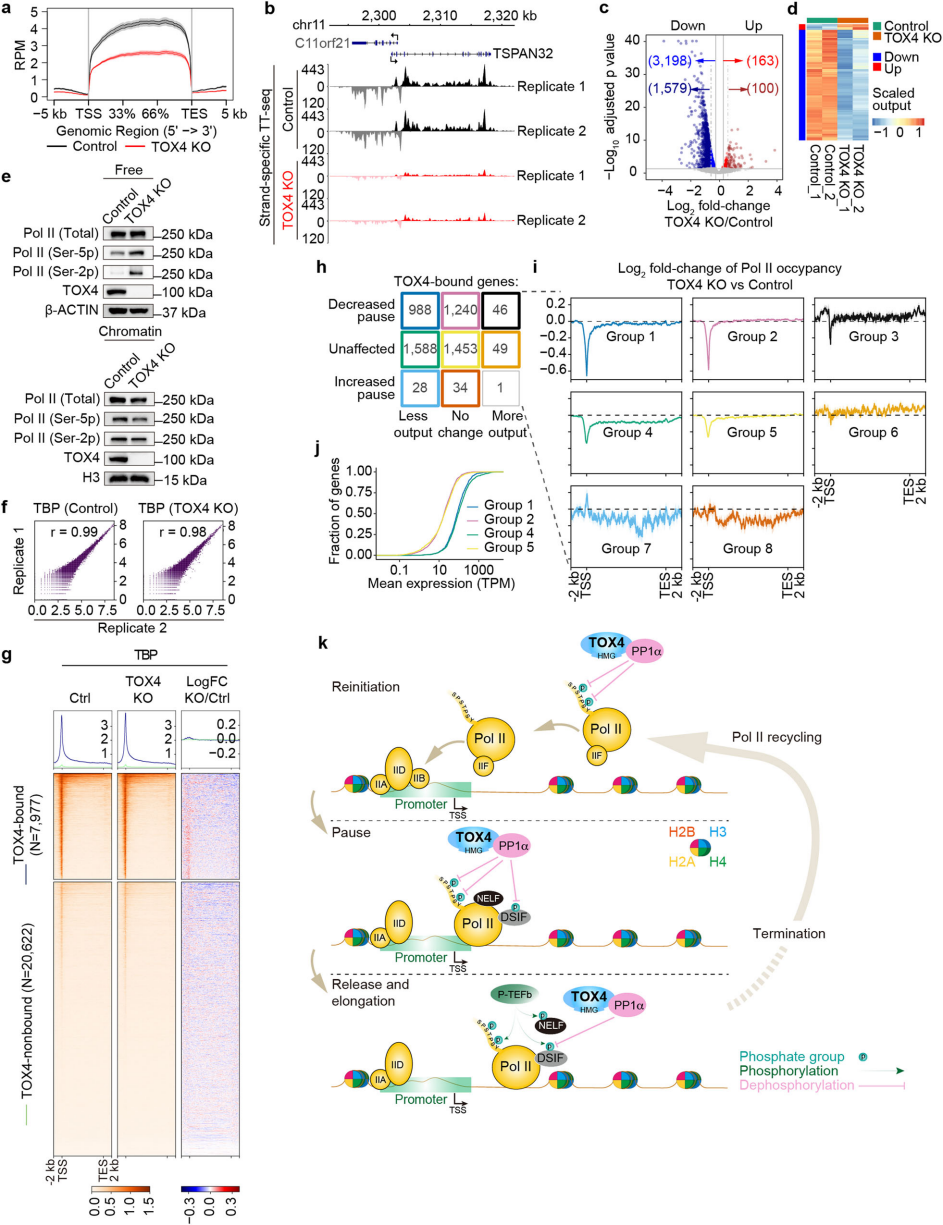
TOX4 promotes Pol II recycling and transcriptional reinitiation through facilitating CTD dephosphorylation. **a** Metagene profiles of TT-seq of protein-coding genes in TOX4 KO versus control cells. **b** Normalized read distribution of TT-seq within the *C11orf21* and *TSPAN32* loci in TOX4 KO versus control cells. **c** A volcano plot of TT-seq showing transcriptional output changes of TOX4-bound genes upon TOX4 loss. **d** A heatmap of TT-seq showing TOX4 direct targets with transcriptional output fold change ≥ 1.2 or ≥ 1.5 upon TOX4 loss. **e** Comparison of levels of free (up) and chromatin-bound (down) total, Ser-5 phosphorylated, and Ser-2 phosphorylated Pol II by Western blot in control and TOX4 KO cells. **f** Correlation plots for biological replicates of CUT&Tag of TBP. **g** Genome-wide meta-gene profiles and heatmaps of CUT&Tag comparing chromatin occupancies of TBP in TOX4 KO versus control cells. Genes were sorted by total Pol II CUT&Tag signal in control cells. **h** Nine groups of genes sorted according to their Pol II TR and transcriptional output status upon TOX4 loss. **i** Pol II metagene profiles of eight out of the nine groups of genes in **h**. **j** Cumulative frequency curves comparing mRNA levels of Groups 1, 2, 4, and 5 genes in **h** in K562 cells. **k** Working model of TOX4 in transcriptional regulation. TOX4 restricts pause release and early productive elongation by facilitating Pol II CTD Ser-2 and SPT5 Thr-806 dephosphorylation, and promotes reinitiation by facilitating dephosphorylation of serines 2 and 5 of Pol II CTD.

As mentioned above, the reduction of Pol II occupancies on genes, in particular around TSSs (Fig. 2f), upon TOX4 loss may also be due to decreased transcriptional initiation and/or initiation to elongation transition besides increased pause release. However, decreased initiation and/or initiation to elongation transition would decrease transcriptional output whereas increased pause release may increase output^35,43^. To assess if TOX4 regulates initiation and/or initiation to elongation transition, we performed transient transcriptome sequencing (TT-seq)^44^ to measure transcriptional output. We found that TOX4 loss mainly decreases outputs of genes (Fig. 7a, b). With fold change ≥ 1.5, the numbers of genes with decreased and increased output were 1579 and 100, respectively, and with fold change ≥ 1.2, the numbers of genes with decreased and increased output were 3198 and 163, respectively (Fig. 7c, d).

CTD phosphatase, FCP1, has been shown playing a critical role in Pol II recycling and transcriptional reinitiation^45^, and its depletion has been found to increase cellular levels of Ser-2 phosphorylated Pol II and, to a lesser degree, Ser-5 phosphorylated Pol II, while decrease total Pol II occupancies on genes^46^. The effects of TOX4 loss on cellular levels of phosphorylated Pol II (Fig. 2a) and total Pol II occupancies (Fig. 2f) greatly resemble those of FCP1 depletion, which, together with the TT-seq results (Fig. 7a–d), suggest that TOX4 may regulate Pol II recycling and reinitiation through CTD serines 2 and 5 dephosphorylation. In addition, we found that TOX4 loss increased levels of free Ser-2 phosphorylated Pol II and Ser-5 phosphorylated Pol II but slightly decreased level of chromatin-bound Pol II (Fig. 7e), which not only greatly resemble effects of FCP1 depletion^46^ but also are in agreement with our CUT&Tag results (Fig. 2g, h), further supporting a role of TOX4 in reinitiation. Moreover, we found that TOX4 loss had no effect on TBP occupancy (Fig. 7f, g), suggesting that TOX4 is more likely to regulate reinitiation rather than initiation.

Both decreased initiation or reinitiation and increased pause release may decrease Pol II TRs. The number of genes exhibiting decreased outputs is far greater than that of genes exhibiting increased outputs (Fig. 7c, d), suggesting that reduced Pol II TRs of thousands of genes upon TOX4 loss (Fig. 3c) are mainly caused by decreased reinitiation rather than increased pause release. To better analyze the underlying causes for the Pol II TR decreases, we first divided TOX4-bound genes into nine groups according to Pol II TR and output status (Fig. 7h). Considering that decreased initiation or reinitiation would decrease Pol II occupancies on promoters and gene bodies, and that increased pause release usually decreases Pol II occupancies on promoters while increases Pol II occupancies on gene bodies, we subsequently generated metagene profiles of Pol II occupancy changes for each group of genes to facilitate the analyses (Fig. 7i). We found that among the three groups of genes with decreased Pol II TR (Groups 1, 2, and 3), genes of Groups 2 and 3 exhibited increased gene body Pol II occupancies besides decreased promoter Pol II occupancies (Fig. 7i), suggesting that increased pause release contributes to Pol II TR decreases of these genes although some of them may also have decreased reinitiation. It is known that increased pause release and/or elongation may increase transcriptional outputs^35,43^. With respect to why genes with increased pause release (Group 2) did not show increased outputs, possible explanations include (1) TOX4 inhibits early elongation but stimulates late productive elongation so that the overall effect on output is small, and (2) upon TOX4 loss, decreased outputs as the results of decreased reinitiation offset increased outputs as the results of increased paused release.

The next question we asked is why among the four groups (Groups 1, 2, 4, and 5) with larger number of genes relative to the rest of the groups (Groups 3, 6, 7, 8, and 9), outputs of Groups 1 and 4 genes are more sensitive to TOX4 loss than those of Groups 2 and 5 genes. Considering that outputs and mRNA level of genes are mainly determined by initiation frequency^35^, we think that it may be due to their higher expression levels relative to those of Groups 2 and 5 genes. This idea was confirmed afterwards by comparison of mRNA levels of these four groups (Fig. 7j).

### TOX4 regulates transcription of a subset of extragenic transcripts

Considering that WDR82, SET1, PNUTS, and PP1 were found to restrict transcription of PROMPT and eRNA by enforcing PA sites-dependent early termination^26^, the last question we asked was if TOX4 regulates transcription of extragenic transcripts. To this end, we further analyzed the TT-seq data. We found that 3813 extragenic transcripts exhibited expression changes upon TOX4 loss, among them, 966 are direct targets of TOX4, and among the direct targets, 593 and 373 were upregulated and downregulated, respectively (Supplementary Fig. 6a, b). In addition, annotation of differentially expressed extragenic transcripts discovered that TOX4 mainly regulates transcription of eRNA, lncRNA, and PROMPT (Supplementary Fig. 6c).

## Discussion

TOX4 is one of the poorly understood regulatory factors of PP1 phosphatases^25^. By analyzing the effects of its loss on Pol II occupancy, CTD phosphorylation and elongation rate of Pol II, chromatin accessibility, PP1 phosphatases recruitment and transcriptional output, we discovered that TOX4 may play three roles in transcription, including facilitating transcriptional reinitiation, restricting pause release and early productive elongation, and promoting late productive elongation (Fig. 7k).

TOX4 was recently found to be one of the subunits of the PP1C, which consists of three regulatory proteins, PNUTS, TOX4, and WDR82, and one of the PP1 phosphatases, PP1α, β, and γ^25^. Within the PP1C, PNUTS was found to be the scaffold and the only subunit that is capable of binding PP1 phosphatases. In contrast, we found that TOX4 is also capable of binding all the three PP1 phosphatases, and it preferentially binds PP1α. In addition, we found that TOX4 facilitates Pol II CTD dephosphorylation by in vitro phosphatase assay. The difference between our study and the previous study^25^ with respect to the PP1 phosphatase binding capability of TOX4 may be caused by the source difference of PP1α used in the two studies, i.e., our PP1α was expressed by and purified from High Five cells and their PP1α was expressed by and purified from *E. coli*.

Pol II CTD dephosphorylation is necessary for Pol II recycling and transcriptional reinitiation, in which FCP1 has been shown to play a critical role^45,46^, but the underlying mechanisms are incompletely understood^18^. The great resemblance of effects of TOX4 KO and FCP1 depletion on Pol II occupancy and CTD phosphorylation, the great impact of TOX4 loss on transcriptional outputs, and unaffected TBP occupancies upon TOX4 loss suggest that TOX4 is another critical regulator of Pol II reinitiation. Moreover, we found that, similar to FCP1^45^, TOX4 is also capable of promoting elongation (Fig. 4f–h). However, future works are needed for further understanding the role of TOX4 in promoting elongation as well as the connections and the differences between FCP1 and TOX4-PP1α.

PNUTS-PP1 has been found restricting elongation and facilitating termination by dephosphorylating DSIF^16,26^. In the current study, TOX4-PP1α was found facilitating reinitiation, restricting early elongation and promoting late productive elongation. These results suggest that PNUTS and TOX4 are functionally non-redundant, and future works are needed to further understand the connections and the differences between them. P-TEFb plays a central role in pause release by phosphorylating DSIF, NELF, and Ser-2 of Pol II CTD^8,36^. The PP2A-Integrator complex has been found recently to regulate multistep of transcription, including restricting pause release and productive elongation by antagonizing Pol II CTD Ser-2 and SPT5 Thr-806 phosphorylation by P-TEFb^11–13^. We found in the current study that TOX4 is also capable of restricting pause release and early productive elongation by facilitating dephosphorylation of the same sites. Future works are needed to understand the connections and the differences between the PP2A-Integrator complex and TOX4-PP1α.

## Methods

### Cells and cell culture

Human cells K562 and 293T were cultured in 90% DMEM + 10% FBS + 2% Penicillin/Streptomycin + 2 mM L-Glutamine. Insect cells SF9 and High Five were cultured in SIM SF medium (Sino Biological. cat. no. MSF1).

### Quantitative immunoblotting

The images were acquired using the ChemiDoc Touch System (Bio-Rad), and the quantification was performed using ImageJ. The primary antibodies used were Pol II (1:1000, sc-899, Santa Cruz), Pol II Ser-2p (1:2000, 61083, Active Motif), Pol II Ser-5p (1:2000, 61085, Active Motif), TOX4 (1:3000, A304–873A, Bethyl), PP1α (1:3000, A300–904A, Bethyl), PP1β (1:3000, A300–905A, Bethyl), PP1γ (1:3000, A300–906A, Bethyl), PNUTS (1:3000, A300–439A, Bethyl), WDR82 (1:2000, 99715, CST), SPT5 (1:3000, A300–868A, Bethyl), and p-SPT5 Thr-806 (1:3000, Fisher Lab).

### Generation of knockout and knockin cell lines by CRISPR-Cas9

Guide RNAs (gRNAs) were designed using the tool provided by Benchling. For the generation of knockout cell lines, a K562-derived cell line, K562-iCas9, inducibly expressing Cas9 was generated by transducing K562 cells with pCW-Cas9-Hygro and selecting clones with high-level expression^47^; gRNAs were cloned into lentiGuide-Puro (Addgene, Plasmid #52963), and individually transduced into K562-iCas9 cells; single colonies obtained by serial dilution were expanded and subsequently characterized by Western blot. For the generation of AID-TOX4 knockin cell line, a K562-derived cell line, K562-TIR1(F74G), expressing TIR1(F74G) was generated by transducing K562 cells with pBabe TIR1-9myc (Addgene, Plasmid #64945) with TIR1 (F74G) mutation and selecting clones with high-level expression; gRNAs were cloned into pSpCas9(BB)-2A-GFP (PX458) (Addgene, Plasmid #48138), and electroporated into K562-TIR1(F74G) cells with linearized donor plasmids; GFP^+^ cells were sorted out by FACS 48 h after electroporation; single colonies obtained by serial dilution were expanded and subsequently characterized by PCR and Western blot. Sequences of gRNAs used in this study are listed in Supplementary Table 1.

### CUT&Tag and data analyses

CUT&Tag experiments were performed as previously described with minor modifications^48^. Briefly, 100,000 cells were used for each experiment. Cells were bound to Concanavalin A-coated beads without fixation and chromatin opening. After primary and secondary antibodies binding, pA-Tn5 transposome binding, and tagmentation, DNA was extracted and amplified by PCR.

Raw reads were filtered using fastp (version 0.13.1, default parameters)^49^ and aligned to hg38 using Bowtie2 (version 2.3.4.1)^50^. Low-quality alignments were filtered out using SAMtools (version 0.1.19)^51^ with command “samtools view -F 1804 -q 25”. MarkDuplicates tools in Picard (http://broadinstitute.github.io/picard/) was used to identify and remove PCR duplicates from the aligned reads. Peak calling was performed using SEACR (version 1.3)^52^ with a top signal threshold 0.01 in stringent mode. Peaks initially were called from merged reads of two biological replicates, and among them, those cannot be called from either of the biological replicates were removed afterwards. The remaining peaks were defined as high confidence peaks. Annotation of peaks was performed using ChIPseeker (version 1.18.0)^53^ package in Bioconductor.

### RNA extraction, reverse transcription, RNA-seq, and data analyses

RNA was extracted from cells using RNeasy Plus Mini Kit (Qiagen, cat. no. 74134) and Quick-RNA MiniPrep Kit (Zymo Research, cat. no. R1054) by following the manufacturers’ protocols. Libraries of strand-specific RNA-seq were constructed as previously described^54^. Raw reads were filtered using fastp (version 0.13.1, default parameters)^49^ and mapped to hg38 using HISAT2 (version 2.1.0)^55^ with parameters “--rna-strandness RF –dta”. Read counts per gene were calculated in strand-specific manner using featureCounts^56^. Differential expression analyses were performed using DESeq2 (version 1.22.2)^57^, and genes with mean TPM ≥ 1, adjusted *P* value < 0.05 and fold change ≥ 1.5 were identified as significantly differentially expressed. Kyoto Encyclopedia of Genes and Genomes (KEGG) pathway enrichment analyses of differentially expressed genes were performed using clusterProfiler (version 3.12.0)^58^.

### ChIP, ChIP-sequencing (ChIP-seq), and data analyses

ChIP assays were performed as previously described^9^. Normally, cells were fixed with 0.4% (v/v) formaldehyde at room temperature for 10 min. To improve the ChIP efficiency, double fixation was used. For double fixation with EGS (Thermo, cat. no. 21565) and formaldehyde, cells were fixed initially with 1.5 mM EGS at room temperature for 30 min, and subsequently with 0.4% formaldehyde at room temperature for 10 min. For sonication, fixed cells were washed twice with PBS and resuspended in ice-cold RIPA-0.3 buffer (10 mM Tris-HCl, 1 mM EDTA, 1% Triton X-100, 0.1% SDS, 0.1% NaDOC, and 0.3 M NaCl, pH 7.4) supplemented with Protease Inhibitor Cocktail (Millipore, cat. no. 535140) at a concentration of 40 million cells/ml; genomic DNA was disrupted to a size range of 100 to 500 bp. For immunoprecipitation, on day 1, antibodies were diluted in RIPA-0.3 and bound to Dynabeads protein A (Thermo, cat. no. 10002D) by incubating at 4 °C for 3 h. Afterwards, the bead-antibody complexes were washed twice with RIPA-0.3 and then incubated with sonicated chromatin at 4 °C overnight. On day 2, after two washes with RIPA-0.3, two washes with RIPA-0, two washes with LiCl buffer (10 mM Tris-HCl, 1 mM EDTA, 0.25 M LiCl, 0.25% NP-40, and 0.25% NaDOC, pH 7.4), and two washes with TE buffer, bound protein-DNA complexes were resuspended in elution buffer (10 mM Tris-HCl, 1 mM EDTA, 0.2 M NaCl, and 1% SDS, pH 7.4) supplemented with 10 µg/ml RNase A for both elution and RNA digestion, and incubated at 55 °C for 1 h. Proteinase K then was added to a final concentration of 200 µg/ml, and after 30 min incubation, the temperature was increased to 65 °C for crosslink reversal. After incubation for 4–6 h, DNA was purified by ChIP DNA Clean & Concentrator (Zymo Research, cat. no. D5205).

ChIP-seq libraries were constructed with 2–10 ng immunoprecipitated DNA. After end-repair, A-tailing, and barcode ligation, barcoded DNA was amplified by 16-cycle to 18-cycle PCR. Raw reads were filtered using fastp (version 0.13.1, default parameters)^49^ and aligned using Bowtie2 (version 2.3.4.1)^50^ to Bowtie2 index based on hg38 downloaded from NCBI. Low-quality alignments were filtered out using SAMtools (version 0.1.19)^51^ with command “samtools view -F 1804 -q 25”. MarkDuplicates tools from Picard was used to identify and remove PCR duplicates from the aligned reads. Peak calling was carried out using MACS2 (version 2.2.6)^59^ with input control. Narrow peaks were called with parameters “-q 0.000001 --nomodel --shift 0 --keep-dup all”.

### 4sUDRB-seq and data analyses

4sUDRB-seq experiments were performed as previously described^34^ with minor modifications. Briefly, 10 million cells were used for each experiment. After DRB (Sigma, cat. no. D1916) treatment and 4sU (Sigma, cat. no. T4509) incorporation, total RNA was extracted. After RNA biotinylation and free biotin removal, biotinylated RNA was purified by streptavidin-coupled Dynabeads (Thermo, cat. no. 11205D). Before library construction, rRNA was depleted by following a published protocol^60^. Sequencing libraries were constructed by following the Illumina TruSeq RNA Library preparation protocol.

4sUDRB-seq reads were filtered using fastp (version 0.13.1, default parameters)^49^ and aligned to the human genome hg19 using Bowtie2 (version 2.3.4.1)^50^ with parameter “-N 1”. Only paired reads aligned to the same chromosome, not to chromosome chrUn_gl000220 (rRNA), and with alignment scores ≥ 5 were kept using awk. BamCoverage from deepTools (version 3.3.1)^61^ was used to generate bigwig files of normalized read coverage per 50-bp bin.

Transcripts were filtered to calculate elongation rates. Specifically, considering that average transcriptional elongation rate in human cells is ~3.8 kb/min, genes with minimum length over 30 or 60 kb, nonoverlapping with other genes, and free of other genes 2 kb upstream of their TSSs were selected for calculating elongation rates from 0 to 10 min or 0 to 20 min. In the end, 8412 genes with length over 30 kb and 4840 genes with length over 60 kb were used to identify elongation boundaries at 10 and 20 min, respectively. Elongation boundaries were identified using a three state Hidden Markov Model (HMM)^62^. In this model, 2 kb regions upstream of TESs were not included because of the unstable signals within them.

Elongation rates were only calculated for genes with clear elongation boundaries in both replicates at each time point in each cell type. Elongation rates from 0 to 10 min or 0 to 20 min were calculated by dividing average Pol II traveling distances (kb) by elongation time (10 or 20 min). Elongation rates from 10 to 20 min was calculated by linearly fitting the averaged 10 min and 20 min boundaries as a function of time. The slope of the linear fit was defined as elongation rate, and the 50% confidence interval of the slope was defined as the confidence interval of the elongation rate. Only genes with positive slope, confidence interval narrower than 0.5 kb/min, and slope intersection with the time axis greater than −10 min were retained.

For defining gene elongation rate changes from 0 to 10 min and 10 to 20 min in TOX4 KO versus control cells, a gene would be considered elongating faster upon TOX4 loss if Pol II travel distance in either biological replicate of TOX4 KO cells is greater than that in either biological replicate of control cells, and conversely, it would be considered elongating slower. Other than those, its rate would be considered unaffected.

### ATAC-seq and data analyses

Tn5 transposase expression and purification, and transposome assembly was conducted as previously described^63^. ATAC-seq experiments were performed by following a published protocol^64^. Briefly, 50,000 cells were used for each experiment. After nuclei preparation, tagmentation, termination, and DNA purification, samples were amplified by PCR with one universal forward primer and different indexed reverse primers.

ATAC-seq pair-end reads were filtered using fastp (version 0.13.1, default parameters)^49^ and aligned to the human genome hg38 using Bowtie2 (version 2.3.4.1)^50^ with parameter “-X 2000”. SAMtools was used to filter reads that mapped to Chr1-22 and ChrX, and MarkDuplicates tool from Picard was used to identify and remove PCR duplicates from the aligned reads. The final deduplicated BAM file was used in the downstream analyses.

Tn5 transposase insertions, which refer to the precise single-base locations where Tn5 transposase accessed the chromatin, were identified by correcting the read start positions by a constant offset (“+” stranded +4 bp, “−” stranded -5 bp). To generate depth-normalized accessibility tracks, bigwig files were constructed based on the Tn5 offset-corrected insertion sites using GenomicRanges^65^ and rtracklayer^66^ packages in R. Meta-gene profile plots were generated using computeMatrix and plotProfile from deepTools^61^. Peak calling was performed on the Tn5-corrected single-base insertions using the “MACS2 callpeak” command with parameters “-g hs -q 0.01 --shift -19 --extsize 38 --nomodel --nolambda --keep-dup all --call-summits”. The peaks were then filtered to remove peaks overlapping the hg38 blacklisted region (http://mitra.stanford.edu/kundaje/akundaje/release/blacklists/hg38-human/hg38.blacklist.bed.gz). Peaks initially were called from merged reads of two biological replicates, and among them, those cannot be called from either of the biological replicates were removed afterwards. The remaining peaks were defined as high confidence ones. A consensus peak set was obtained by merging the high confidence peaks identified in each cell type using mergeBed. Peak annotation of high confidence peaks of each cell type and the final consensus peak set was performed using ChIPseeker (version 1.18.0)^53^ package in Bioconductor.

Tn5 transposase insertion count matrix was constructed by counting Tn5 transposase insertions in each consensus peak in every sample, and was taken as input for edgeR^67^ to perform differential accessibility analysis. Consensus peaks with FDR-adjusted *P* value < 0.01 and fold change ≥ 2 were defined as differentially accessible peaks upon TOX4 loss, and differentially accessible TOX4-binding sites are those differentially accessible peaks with TOX4 occupancy. Differential accessibility analysis for gene body regions (from 2 kb downstream of TSS to TES) was performed as described above with minor modifications, and gene body regions with FDR-adjusted *P* value < 0.05 and fold change ≥ 1.2 were defined as differentially accessible.

### TT-seq and data analyses

TT-seq experiments were performed as previously described^44,68^ with minor modifications. Briefly, 3.5 × 10^7^ cells were used for each experiment; cells were transferred to fresh antibiotics-free medium and cultured for 1 h before 4-thiouridine (4sU) treatment; total RNA was extracted using TRNzol according to the manufacturer’s instructions; 1 μg 4-thiouracil (4TU) labeled *S. cerevisiae* BY4741 RNA was added to 100 μg total human RNA as spike-in; After fragmentation, biotinylation of 4sU-labeled RNA, purification of biotinylated RNA with Dynabeads M-280 streptavidin and rRNA depletion, strand-specific RNA-seq libraries were constructed as previously described^54^.

Analyses of TT-seq data were performed as previously described^68^ with minor modifications. Specifically, raw reads were filtered using fastp (version 0.13.1, default parameters)^49^. For target (*Homo sapiens* GRCh38) or spike-in (*S. cerevisiae* sacCer3) genome, genome sequences and RefGene annotation file were downloaded from UCSC, and STAR genome index was prepared using STAR (version 2.7.9a)^69^ with the “--runMode genomeGenerate” option. Filtered reads were aligned against each index using STAR with the “-quantMode GeneCounts” option. SAMtools (version 0.1.19)^51^ and Picard were used to sort, index, and mark duplicate reads in the resulting genome BAM files.

Yeast and human gene count matrixes each were constructed from the STAR output files. The yeast gene count matrix was passed to the “estimateSizeFactors” function in the Bioconductor DESeq2 package^57^ to calculate a scale factor for each individual sequencing sample. The human gene count matrix, along with the scale factors of all samples, was used in differential expression analysis with DESeq2, and genes with mean CPM ≥ 1, adjusted *P* value < 0.05 and fold change ≥ 1.2 were identified as significantly differentially expressed. KEGG pathway enrichment analyses of differentially expressed TOX4-bound genes were performed using clusterProfiler (version 3.12.0)^58^. To create scaled, strand-specific BigWig files, SAMtools was used to split each target BAM file into two files containing mapped reads on either plus or minus strand, and bamCoverage function of deepTools was used with the “-scaleFactor” argument to convert each strand-specific BAM file to a scaled BigWig file. To create sense or antisense meta-profiles for gene-body regions of TOX4-bound genes, mate 2 reads in the BAM files were selected using SAMtools and passed to Ngs.plot using the “-SS” option.

### Co-immunoprecipitation

Co-IP assays were performed as previously described^70^ with minor modifications. Nuclear extract (NE) from K562 cells was diluted 2-fold to 3-fold by adding NE dilution buffer. Antibodies were incubated with Dynabeads protein A at 4 °C for 3 h, and then cross-linked to beads by 25 mM DMA (Pierce, cat. no. 20660) at room temperature for 1 h. Normally, 0.5 to 1 mg nuclear extract was used for each co-IP. After overnight incubation at 4 °C, bead-antibody-protein complexes were washed 5 times with BC-200 buffer (10 mM Tris-HCl, 0.2 mM EDTA, 200 mM KCl, 20% glycerol, and 0.1% NP-40, pH 7.9). Proteins were eluted from beads with 50 mM glycine (pH 2.4), immediately neutralized in 1 M Tris-HCl, pH 7.4, and analyzed by Western blot.

### Protein expression and purification

For purifying TOX4, PP1α, PP1β, and PP1γ, cDNAs of 3× Flag-tagged TOX4 and His-tagged PP1α, β, and γ were cloned into pFASTBAC.1 (Invitrogen), respectively. Bacmids were extracted from DH10Bac *E. coli* transformed with pFASTBAC1-3× Flag-TOX4, pFASTBAC.1-His-PP1α, β, or γ. Bacmids were transfected into SF9 cells for baculovirus production by following the manufacturer’s protocol. High Five cells were used for protein expression and purification. After infected by baculoviruses for 72 h, High Five cells were homogenized in BC-300 buffer supplemented with protease inhibitors (0.5 μg/ml pepstatin and 0.5 μg/ml leupeptin) until more than 90% of the cells were lysed (monitored microscopically by trypan blue staining). Lysate was incubated on ice for 30 min followed by centrifugation at 30,000 rpm for 20 min or more to remove cell debris. Anti-Flag M2 Magnetic beads (Sigma, ca. no. M8823) were added to soluble extract for the purification of TOX4, rotated at 4 °C for 3 h, and washed three times with BC-400 buffer 0.05% NP-40. Protein complexes were eluted by BC-300 buffer containing and 0.5 mg/ml 3× Flag peptide. HisPur^TM^ Ni-NTA Magnetic beads (Thermo, ca. no. 88831) were added to soluble extract for the purification of PP1α, β, or γ, rotated at 4 °C for 3 h, and washed five times with wash buffer (300 mM NaCl, 10 mM Tris-HCl, 50 mM imidazole, 0.05% Tween-20). Proteins were eluted by elution buffer (300 mM NaCl, 10 mM Tris-HCl, 500 mM imidazole).

Human Pol II was purified from 293T cells stably expressing 3× Flag-Rpb3. Briefly, cells were cultured in 293 serum-free medium (Union-Biotech, cat. no. UP1000) in a shaker at 37 °C and harvested at the density of 4 × 10^6^ cell/ml. Cells from ~400 ml medium were collected and washed with PBS. Cell pellet was resuspended in 100 ml of hypotonic buffer (10 mM Tris-HCl 7.5, 1.5 mM MgCl_2_, and 10 mM KCl) supplemented with 1 mM DTT and 1 mM PMSF and placed on ice for 10 min for the release of cytoplasmic proteins. After centrifugation, nuclear pellet was resuspended in high salt buffer (50 mM Tris-HCl 7.5, 1 mM MgCl_2_, 300 mM KCl, 1 mM EDTA, and 1% NP-40) supplemented with 1 mM DTT, 1 mM PMSF, and a complete set of proteinase inhibitors, treated with 75 μl of 10 mg/ml heparin (Sigma, cat. no. h3149) and 1 μl of Benzonase Nuclease (Sigma, cat. no. E1014-5KU) to facilitate Pol II release from genomic DNA and then rotated at 4 °C for 2 h. Insoluble fractions were removed by centrifugation at 13,000 rpm for 1.5 h at 4 °C. The soluble faction was incubated with 100 μl of anti-Flag M2 beads for 4 h at 4 °C. The beads were then washed three times with high salt buffer and eluted with 300 μl of 500 μg/ml of 3× Flag peptides in Flag elution buffer (50 mM Tris-HCl 7.5, 1.5 mM MgCl_2_, 100 mM KCl, 1% NP-40, and 10% glycerol). The eluted proteins were concentrated by a Millipore Ultraconcentrator with MWCO of 3 kDa.

### In vitro phosphatase assay

Pol II was purified from 293T cells, and PP1α, TOX4 and TOX4-PP1α were purified from High Five cells infected by the corresponding recombinant baculovirus. Pol II was incubated alone or with TOX4, PP1α and TOX4-PP1α, respectively, at 30 °C for 1 h in PP1 reaction buffer (50 mM HEPES, 0.1 mM EDTA, 5 mM DTT, 0.025% Tween-20, and 1 mM MnCl_2_, pH 7.5)^25^. The results were analyzed by Western blot.

### Data reproducibility assessment

Reproducibility of two biological replicates or correlation between data of ChIP-seq and CUT&Tag was assessed using Pearson correlation coefficient calculated by multiBamSummary and plotCorrelation function of deepTools^61^. Biological replicates with Pearson correlation coefficient (*r*) greater than 0.90 are considered highly reproducible. The *r* value of PP1β CUT&Tag in control and TOX4 KO cells is lower than 0.90 because the antibody is not very good for CUT&Tag. Highly reproducible biological replicates of ChIP-seq, ATAC-seq or CUT&Tag were pooled together for maximum coverage.

### Read count quantification and analysis

For ChIP-seq, CUT&Tag, and ATAC-seq, bamCompare from deepTools^61^ were used to analyze log_2_ fold changes of read density upon TOX4 loss and create a BigWig file for the changes with “--operation log2 --normalizeUsing CPM –pseudocount 1” options, and computeMatrix and plotProfile/plotHeatmap from deepTools were used to perform metaplot and heatmap analyses of read density or the log_2_ fold changes of read density within regions of interest. Relative changes of read density within regions of interest, for example, occupancy changes of Ser-2p relative to those of Pol II, were calculated using custom R scripts with read density change matrixes taken from outputs of computeMatrix and visualized using plotProfile/plotHeatmap.

For analyzing correlation between mRNA levels and TOX4 binding levels of TOX4 direct targets, all human genes were divided into four groups by their mRNA levels in control K562 cells. Specifically, genes with averaged TPM less than 1 were classified as “Silent”; for the non-silent genes, those with averaged TPM smaller than the first quartile of the average TPM were classified as “Low”, those with averaged TPM greater than the third quartile of the average TPM values were classified as “High”, and the rest of them were classified as “Medium”. Given that only 11 direct targets of TOX4 were allocated into the “Silent” group, we therefore relocated them into the “Low” group to simplify the related analyses. Meta-profiles and heatmaps were generated as previously described^47^.

### Traveling ratio calculation

For each TOX4-bound gene, Pol II CUT&Tag read density (RPKM) on promoter or gene body was calculated as read count normalized by length and sequencing depth, and traveling ratio was calculated as promoter read density divided by gene body read density for genes with promoter read density greater than 0.005. Genes with Pol II TR fold change ≥ 1.5 are considered to be meaningfully affected.

### Identification of differentially expressed extragenic transcripts

In order to identify differentially expressed extragenic transcripts upon *TOX4* depletion, we used SICER^71^ to detect extragenic transcripts according to TT-seq data. Stranded mapped reads were first extracted by SAMtools to obtain files containing reads of plus and minus strands, respectively^51^. Reads overlapping with protein-coding gene were excluded afterwards using BEDTools^72^. Reads of plus or minus strand were analyzed by SICER separately with the following parameters: --window_size 500 --fragment_size 0 --effective_genome_fraction 1 --gap_size 1000 --e_value 100. Regions detected in control and knockout samples were then merged using BEDTools, and only merged regions (hereafter termed extragenic transcripts) with at least 50 reads in each biological replicate of control or knockout were kept for further analyses.

Differential expression analyses of extragenic transcripts upon TOX4 KO were performed using DESeq2^57^ with the sizeFactor set as scale factors calculated from yeast gene matrix. Extragenic transcripts with adjusted *P* value < 0.05 and fold change ≥ 1.5 were identified as significantly differentially expressed.

Extragenic transcripts were annotated by comparative analysis of extragenic transcripts and known genomic features. Priority was given sequentially to Gencode lncRNAs, enhancers and antisense transcript of protein coding genes, and the remaining regions subsequently were annotated to four classes: (1) promoter-upstream transcripts (PROMPTs): within 2 kb of annotated TSSs of RefGene genes and with direction opposite to those of the corresponding mRNAs; (2) readthrough: within 2 kb of annotated TESs of RefGene genes and with direction the same as that of the corresponding mRNAs; (3) promoter convergent: within 2 kb of annotated TSSs of RefGene genes and with direction the same as that of the corresponding mRNAs; (4) other intergenic transcripts: cannot be placed within the categories listed above.

### Statistics and reproducibility

No statistical methods were used to predetermine sample size. The experiments were not randomized, and investigators were not blinded to allocation during experiments and outcome assessment.

All statistical tests were two-sided unless otherwise stated. All bar graphs are representative of three or more independent experiments as indicated in the figure legends. Statistical significance was determined with a two-sided Student’s *t*-test; the centers and the error bars represent the mean and SD, respectively. Where *P* values are reported, an alpha level < 0.05 was considered statistically significant. Two-sided Student’s *t*-test was performed using GraphPad Prism (version 7). Hypergenometric test, Chi-square test and Kolmogorov–Smirnov test were performed by R.

The Benjamini–Hochberg (BH) correction method was used to adjust the *P* values to control FDR where multi-testing corrections were involved. FDR-adjusted *P* values and fold changes (FCs) for differential expression were derived from DESeq2 analysis^57^. *P* values and FCs for differential accessibility were derived from edgeR^67^ analysis, and *P* values were adjusted as mentioned above. Reproducibility of two biological replicates of ChIP-seq, CUT&Tag, or ATAC-seq data were assessed using Pearson correlation coefficient calculated by deepTools. FDR-adjusted *P* values for pathway enrichment were derived from enrichKEGG analysis by clusterProfiler.

### Reporting summary

Further information on research design is available in the Nature Research Reporting Summary linked to this article.

## Supplementary information

Peer Review File

Supplementary Information

Description of Additional Supplementary Files

Supplementary Data 1

Reporting Summary

## Data Availability

Next generation sequencing data have been submitted to GEO repository under accession number GSE164277. The uncropped images of Western blot experiments are available in Supplementary Figs. 7–15. The source data of bar graphs are stored in Supplementary Data 1 file. All other data are available from the corresponding author upon reasonable request.

## Code availability

Details of publicly available software used in the study are given in the “Methods”. No custom code or mathematical algorithm that is deemed central to the conclusions was used.
