# Supplementary material for: TOX4 facilitates promoter-proximal pausing and C-terminal domain dephosphorylation of RNA polymerase II in human cells
Source: Commun Biol. 2022 Apr 1;5:300. doi: 10.1038/s42003-022-03214-1 (PMC8975821; doi:10.1038/s42003-022-03214-1)
Supplement: Supplementary file 2 — Supplementary Information [file 42003_2022_3214_MOESM2_ESM.pdf]

Supplementary Table

Supplementary Table 1. Guide RNAs.

Name	Sequence	Note
**TOX4 Forward**	CACCGCAGTTTTTCAGCCCAGTAT	For TOX4 knockout
**TOX4 Reverse**	AAACATACTGGGCTGAAAAACTGC	For TOX4 knockout
**TOX4 Forward**	CGATGAGGGTCTGAGACGGT	For AID knockin
**TOX4 Reverse**	ACCGTCTCAGACCCTCATCG	For AID knockin

Supplementary Figures

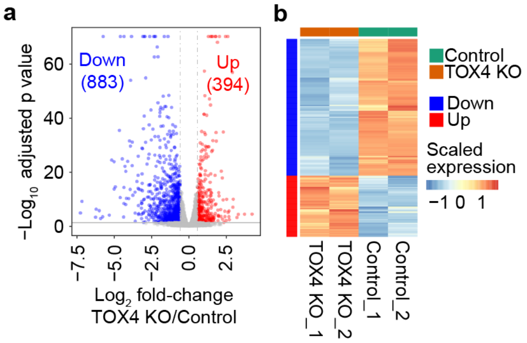

Supplementary Fig. 1. Analyses of gene expression changes upon TOX4 loss in K562 cells by RNA-seq. (a) A volcano plot showing gene expression changes upon TOX4 loss. (b) A heatmap of significantly differentially expressed genes in TOX4 KO versus control cells.

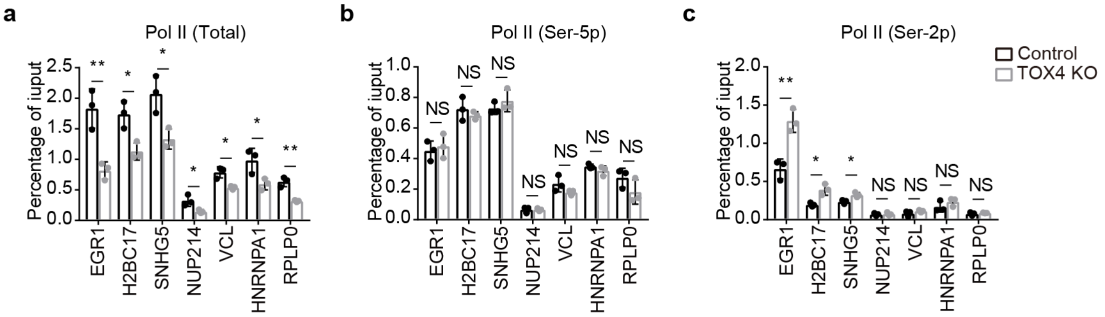

Supplementary Fig. 2. TOX4 loss affects the chromatin occupancy of Pol II. (a), (b) and (c) Analyses of occupancies of total (a), Ser-5 phosphorylated (b) and Ser-2 phosphorylated (c) Pol II by ChIP-qPCR on a number of genes in control and TOX4 KO cells. Statistical significance was determined with a two-sided Student's t-test; the centers and error bars represent the mean and the SD of three biological repeats, respectively. NS: P ≥ 0.05, *P < 0.05, **P < 0.01.

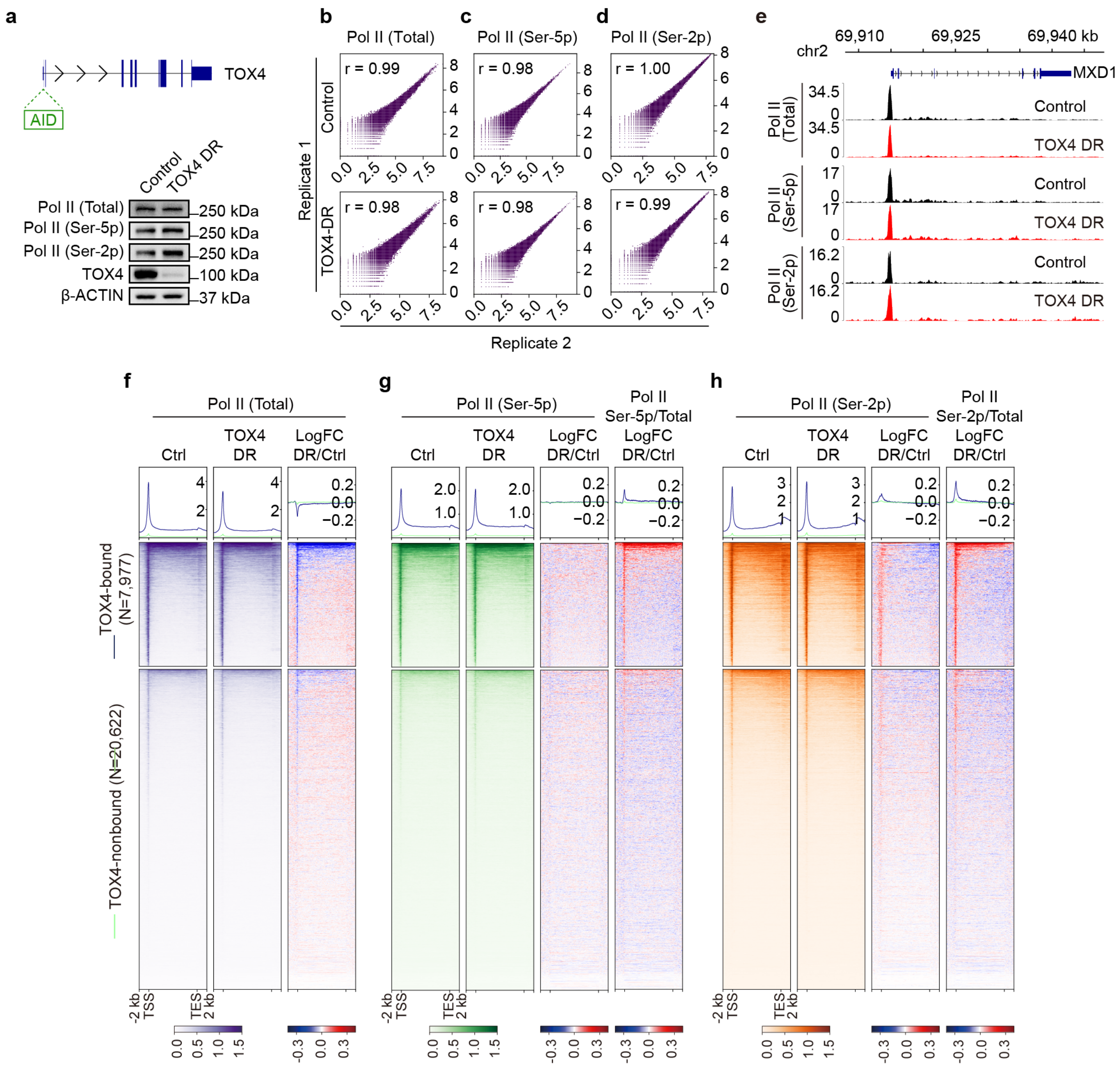
a
TOX4
AID
Control
TOX4 DR
Pol II (Total)
Pol II (Ser-5p)
Pol II (Ser-2p)
TOX4
β-ACTIN
250 kDa
250 kDa
250 kDa
100 kDa
37 kDa
b
Pol II (Total)
c
Pol II (Ser-5p)
d
Pol II (Ser-2p)
Replicate 1
Control
TOX4-DR
r = 0.99
r = 0.98
r = 1.00
r = 0.98
r = 0.98
r = 0.99
Replicate 2
e
chr2
69,910
69,925
69,940 kb
MXD1
Pol II (Total)
Pol II (Ser-5p)
Pol II (Ser-2p)
Control
TOX4 DR
34.5
17
16.2
f
Pol II (Total)
g
Pol II (Ser-5p)
Pol II Ser-5p/Total
h
Pol II (Ser-2p)
Pol II Ser-2p/Total
Ctrl
TOX4 DR
LogFC DR/Ctrl
TOX4-bound (N=7,977)
TOX4-nonbound (N=20,622)
-2 kb
TSS
TES
2 kb

Supplementary Fig. 3. TOX4 downregulation increases CTD phosphorylation and decreases chromatin occupancy of Pol II. (a) Generation and characterization of a cell line with TOX4 downregulation (DR). Top: Schematic of knockin strategy of AID-TOX4. Bottom: Comparison of cellular levels of total, Ser-5 phosphorylated and Ser-2 phosphorylated Pol II by Western blot in control and cells with TOX4 DR. (b), (c) and (d) Correlation plots for biological replicates of CUT&Tag of total (b), Ser-5 phosphorylated (c) and Ser-2 phosphorylated (d) Pol II. (e) Normalized read distribution of CUT&Tag of total, Ser-5 phosphorylated and Ser-2 phosphorylated Pol II within the *MXD1* locus in TOX4 DR versus control cells. (f), (g) and (h) Genome-wide meta-gene profiles and heatmaps of CUT&Tag comparing chromatin occupancies of total (f), Ser-5 phosphorylated (g) and Ser-2 phosphorylated (h) Pol II in TOX4 DR versus control (Ctrl) cells. Genes were sorted by total Pol II CUT&Tag signal in control cells.

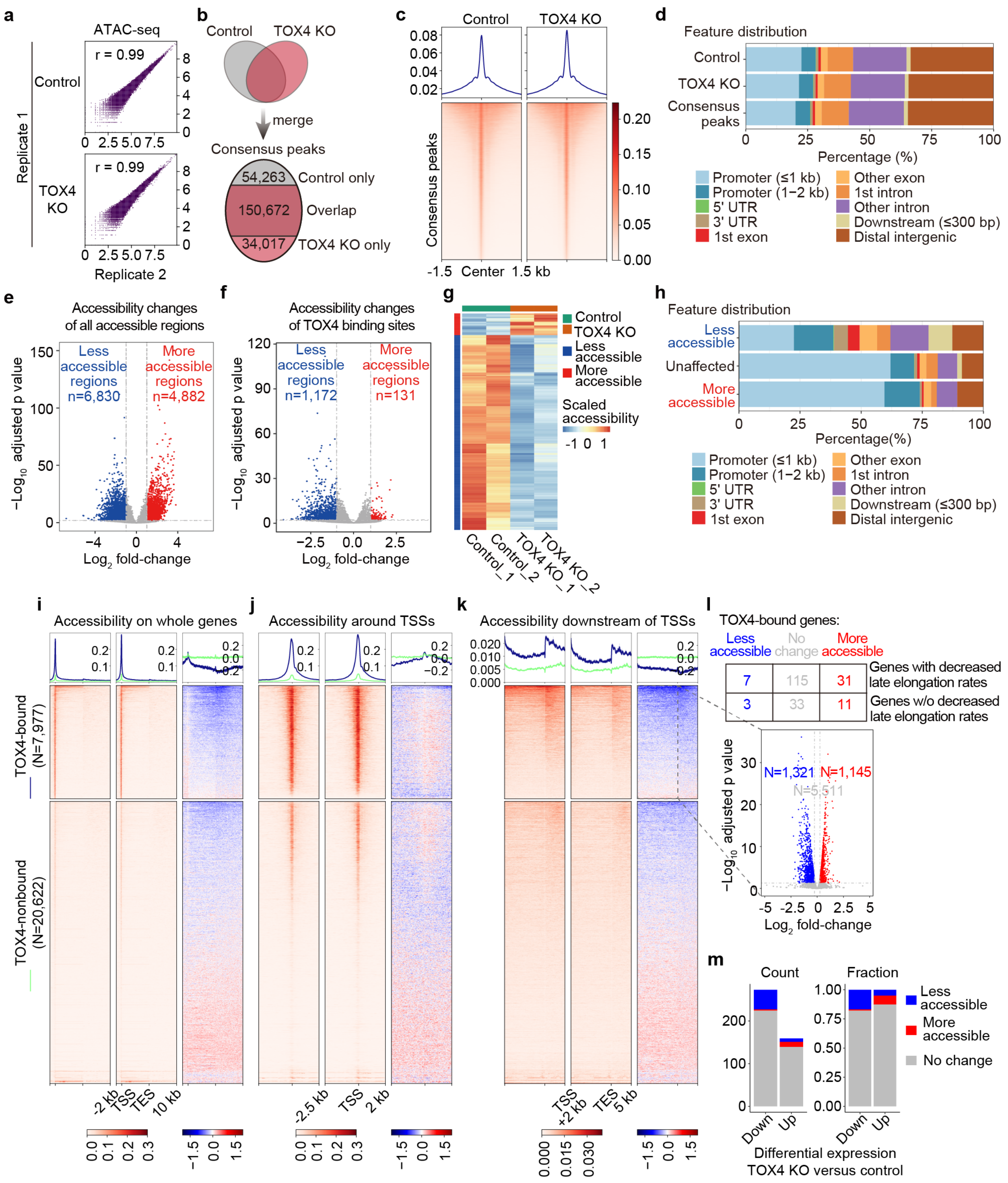

	Less accessible	No change	More accessible
Genes with decreased late elongation rates	7	115	31
Genes w/o decreased late elongation rates	3	33	11

Supplementary Fig. 4. TOX4 may not promote late productive elongation by increasing chromatin accessibility.

(a) Correlation plots for biological replicates of ATAC-seq in control (top) and TOX4 KO (bottom) cells. (b) A diagram describing the obtainment of consensus peaks by merging peaks of control and TOX4 KO cells. (c) Chromatin accessibility near consensus peak center in control and TOX4 KO cells. (d) Genomic annotation of peaks in control cells, peaks in TOX4 KO cells and consensus peaks. (e) A volcano plot showing chromatin accessibility changes upon TOX4 loss. (f) A volcano plot showing accessibility changes of TOX4 binding sites upon TOX4 loss. (g) A heatmap showing TOX4 binding sites with significant accessibility change in TOX4 KO versus control cells. (h) Comparison of genomic distribution of less accessible, unaffected and more accessible sites with TOX4 occupancies in TOX4 KO versus control cells. (i), (j) and (k) Meta-gene profiles and heatmaps of ATAC-seq data comparing chromatin accessibility of genes (i), promoters (j) and gene bodies (k) in TOX4 KO versus control cells. (l) Examination for gene body accessibility changes of the 200 nonoverlapping TOX4-bound genes with lengths over 60 kb and showing clear front borders of elongation. Top: intersection of gene sets grouped by gene body accessibility changes and gene sets grouped by Pol II elongation rate changes, Bottom: a volcano plot showing gene body accessibility changes of all TOX4-bound genes upon TOX4 loss. (m) Stacked bar graphs showing accessibility changes of TOX4 direct targets.

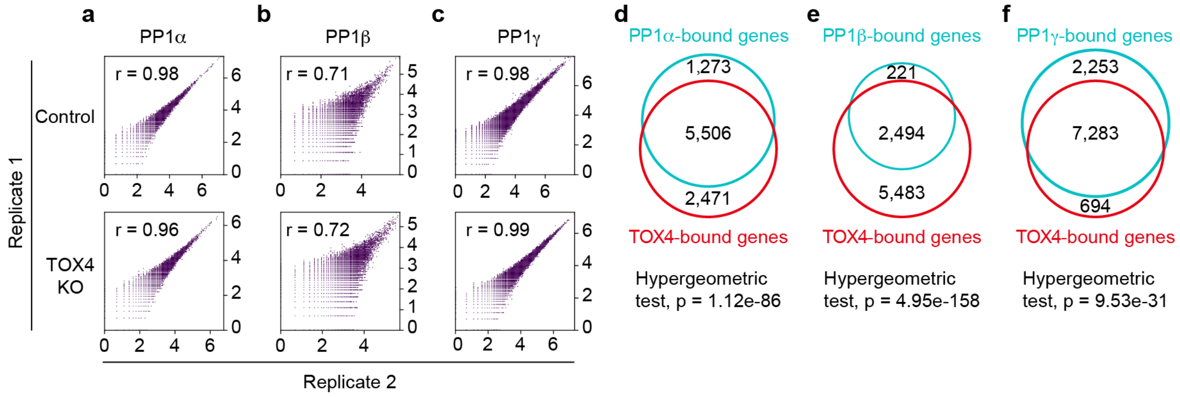

Supplementary Fig. 5. TOX4 co-localizes with PP1 phosphatases on chromatin.

(a), (b) and (c) Correlation plots for biological replicates of CUT&Tag of PP1α (a), β (b) and γ (c) in control and TOX4 KO cells. (d), (e) and (f) Venn diagrams showing overlaps between occupancies of TOX4 and PP1α (d), β (e) and γ (f). Statistical significance of overlap between gene sets was determined with a Hypergeometric test.

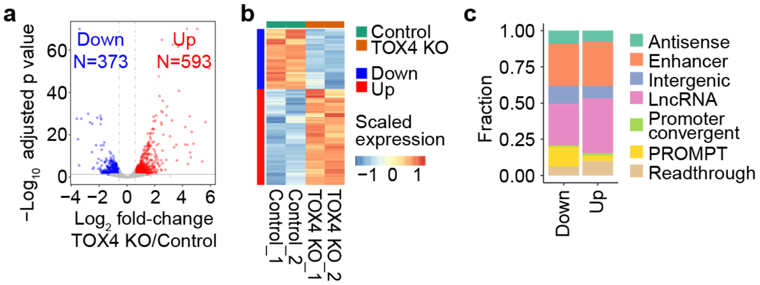

Supplementary Fig. 6. TOX4 regulates transcription of extragenic transcripts.

(a) A volcano plot showing expression changes of TOX4-bound extragenic transcripts upon TOX4 loss. (b) A Heatmap showing TOX4-bound extragenic transcripts with significant expression changes upon TOX4 loss. (c) A stacked bar graph showing annotation of significantly down- and up-regulated TOX4-bound extragenic transcripts upon TOX4 loss.

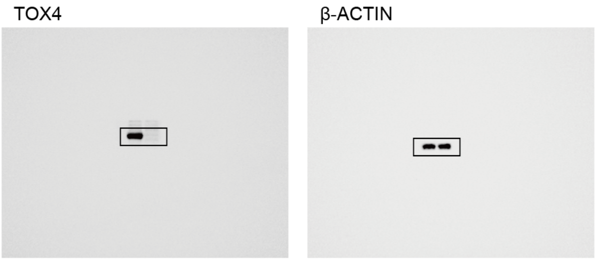

Supplementary Fig. 7. Uncropped blots referring to Fig. 1d.

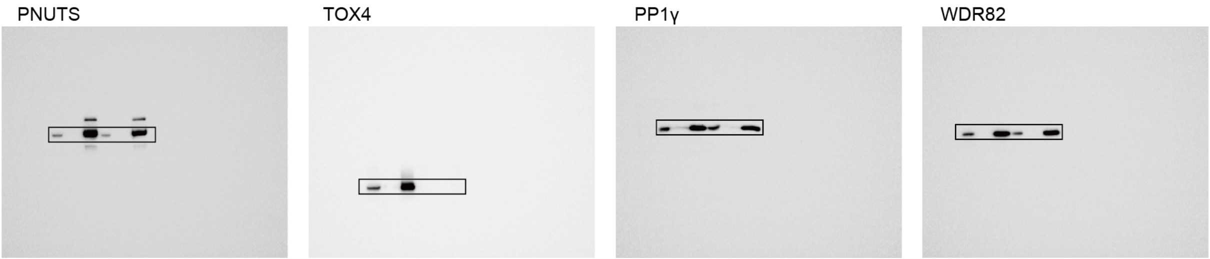

Supplementary Fig. 8. Uncropped blots referring to Fig. 1f.

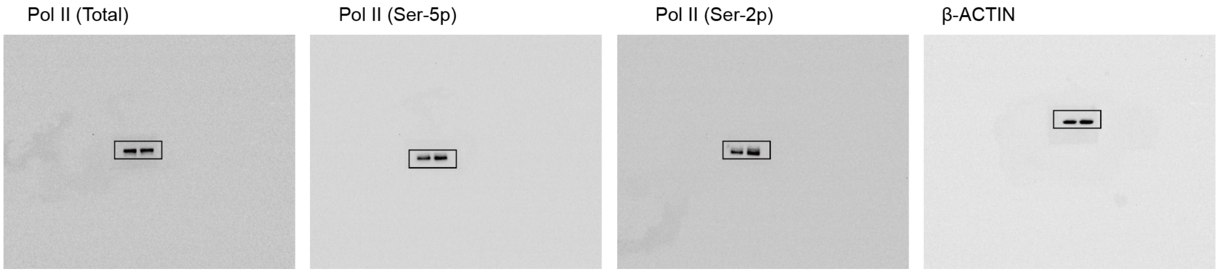

Supplementary Fig. 9. Uncropped blots referring to Fig. 2a.

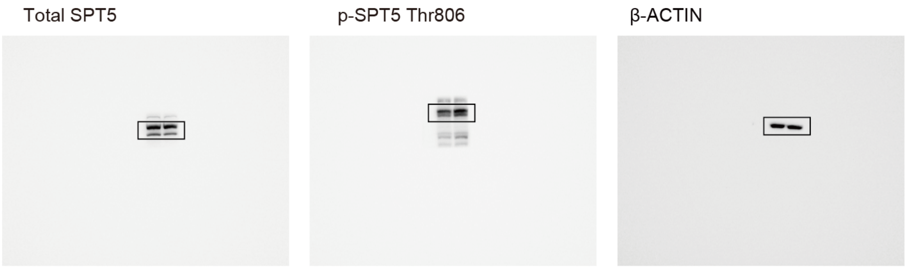

Supplementary Fig. 10. Uncropped blots referring to Fig. 5a.

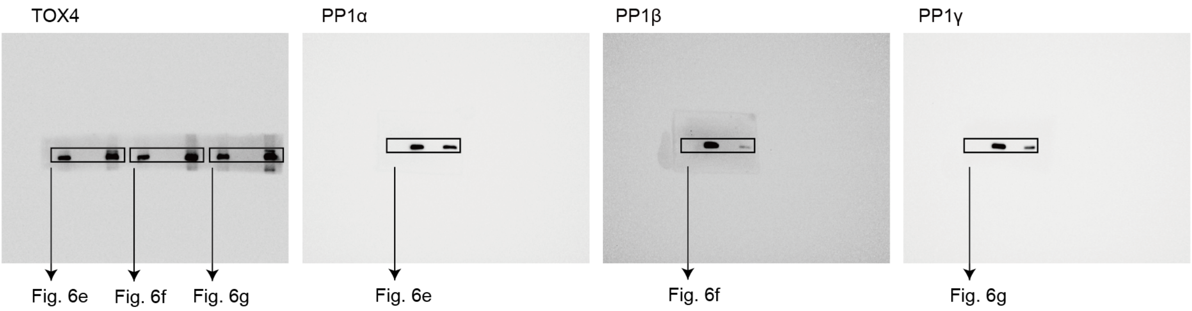

Supplementary Fig. 11. Uncropped blots referring to Fig. 6e-g.

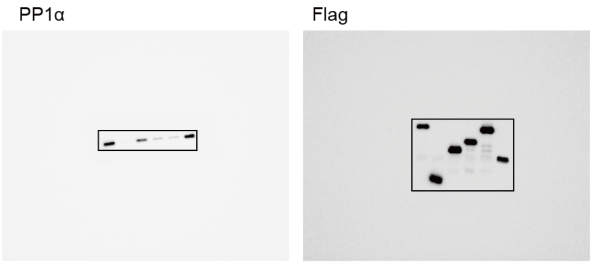

Supplementary Fig. 12. Uncropped blots referring to Fig. 6i.

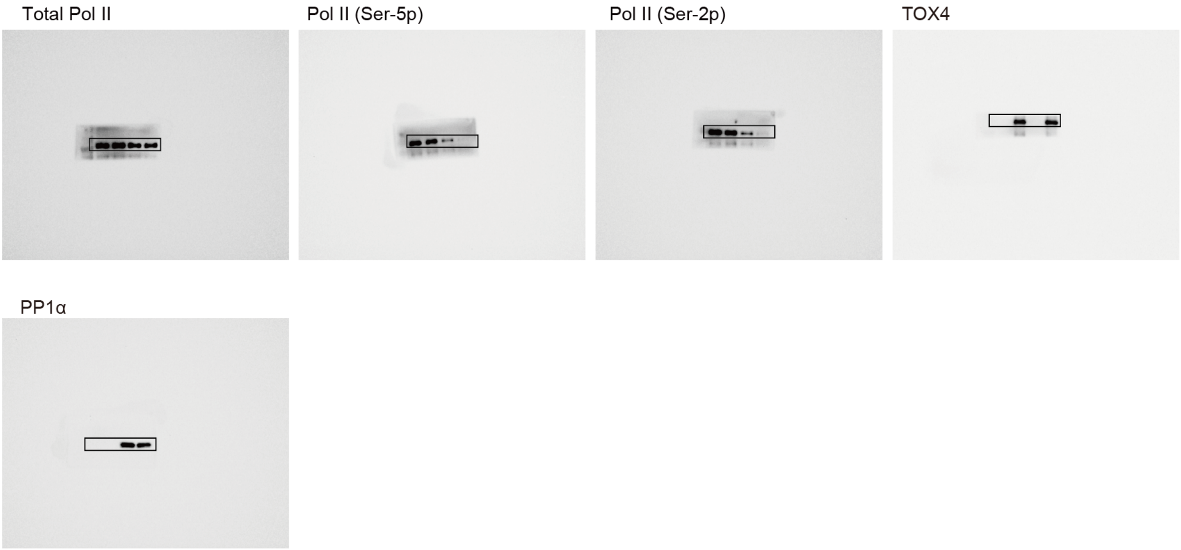

Supplementary Fig. 13. Uncropped blots referring to Fig. 6j.

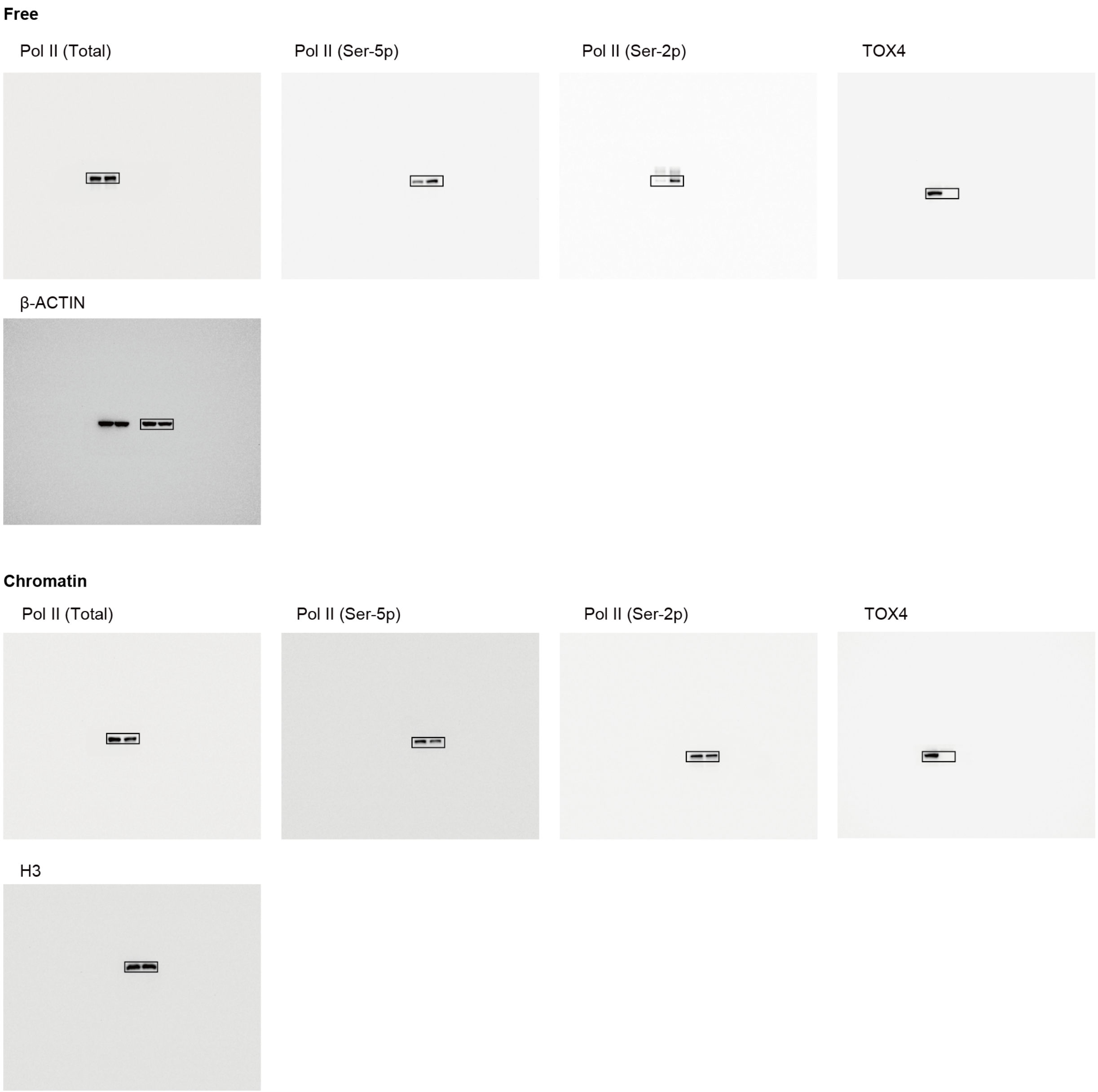

Supplementary Fig. 14. Uncropped blots referring to Fig. 7e.

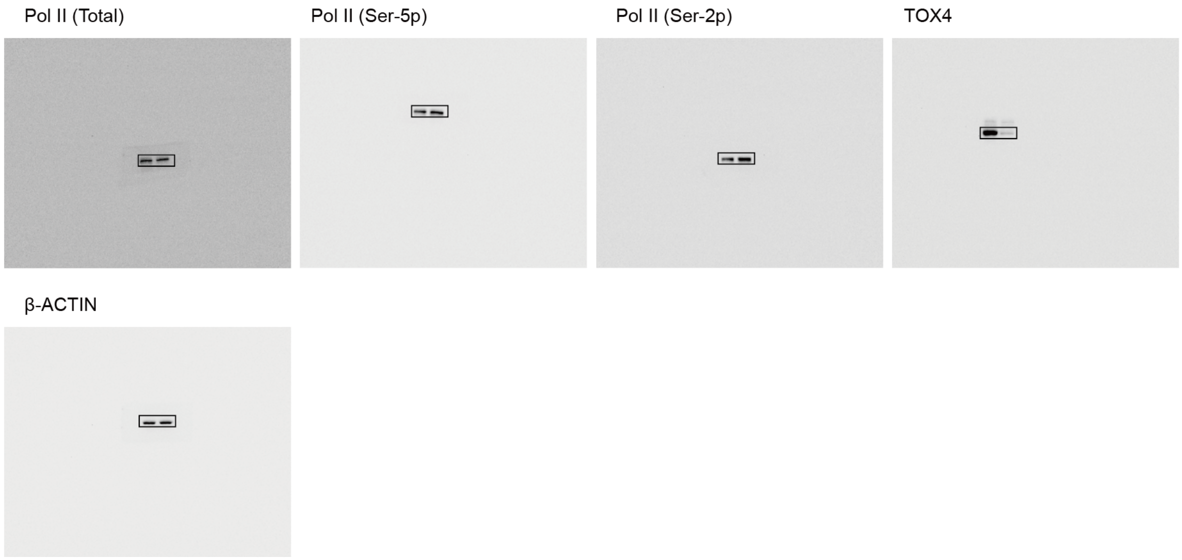

Supplementary Fig. 15. Uncropped blots referring to Fig. S3e.
