# Supplementary material for: TOX4 facilitates promoter-proximal pausing and C-terminal domain dephosphorylation of RNA polymerase II in human cells
Source: Commun Biol. 2022 Apr 1;5:300. doi: 10.1038/s42003-022-03214-1 (PMC8975821; doi:10.1038/s42003-022-03214-1)
Supplement: Supplementary file 3 — Description of Additional Supplementary Files [file 42003_2022_3214_MOESM3_ESM.pdf]

Description of Additional Supplementary Files

File name: Supplementary Data 1
Description: Source data of bar graphs shown in the Figures.
