# Supplementary material for: TOX4 facilitates promoter-proximal pausing and C-terminal domain dephosphorylation of RNA polymerase II in human cells
Source: Commun Biol. 2022 Apr 1;5:300. doi: 10.1038/s42003-022-03214-1 (PMC8975821; doi:10.1038/s42003-022-03214-1)
Supplement: Supplementary file 5 — Reporting Summary [file 42003_2022_3214_MOESM5_ESM.pdf]

nature portfolio

Corresponding author(s): Ming Yu

Last updated by author(s): Jan 30, 2022

Reporting Summary

Nature Portfolio wishes to improve the reproducibility of the work that we publish. This form provides structure for consistency and transparency in reporting. For further information on Nature Portfolio policies, see our Editorial Policies and the Editorial Policy Checklist.

Statistics

For all statistical analyses, confirm that the following items are present in the figure legend, table legend, main text, or Methods section.

n/a	Confirmed	
☐	☒	The exact sample size (*n*) for each experimental group/condition, given as a discrete number and unit of measurement
☐	☒	A statement on whether measurements were taken from distinct samples or whether the same sample was measured repeatedly
☐	☒	The statistical test(s) used AND whether they are one- or two-sided<br>*Only common tests should be described solely by name; describe more complex techniques in the Methods section.*
☒	☐	A description of all covariates tested
☐	☒	A description of any assumptions or corrections, such as tests of normality and adjustment for multiple comparisons
☐	☒	A full description of the statistical parameters including central tendency (e.g. means) or other basic estimates (e.g. regression coefficient) AND variation (e.g. standard deviation) or associated estimates of uncertainty (e.g. confidence intervals)
☐	☒	For null hypothesis testing, the test statistic (e.g. *F*, *t*, *r*) with confidence intervals, effect sizes, degrees of freedom and *P* value noted<br>*Give P values as exact values whenever suitable.*
☒	☐	For Bayesian analysis, information on the choice of priors and Markov chain Monte Carlo settings
☒	☐	For hierarchical and complex designs, identification of the appropriate level for tests and full reporting of outcomes
☐	☒	Estimates of effect sizes (e.g. Cohen's *d*, Pearson's *r*), indicating how they were calculated

Our web collection on statistics for biologists contains articles on many of the points above.

Software and code

Policy information about availability of computer code

Data collection

No software was used for the data collection.

Data analysis

CUT&Tag and ChIP-seq
Raw reads were filtered using fastp (v0.13.1) and subsequently aligned to the human genome using Bowtie2 (v2.3.4.1). Aligned reads were filtered using Samtools (v1.9) and duplicates were removed with Picard. For CUT&Tag, peak calling was performed using SEACR (v1.3), and for ChIP-seq, peak calling was performed using MACS2 (v2.2.6). Annotation of peaks was performed using ChIPseeker (v1.18.0).

4sUDRB-seq
Raw reads were filtered using fastp (v0.13.1) and subsequently aligned to the human genome using Bowtie2 (v2.3.4.1). Aligned reads were filtered using SAMtools (v1.9). Elongation boundaries were identified using a three state Hidden Markov Model (HMM)(Danko et al., 2013).

ATAC-seq
Raw reads were filtered using fastp (v0.13.1) and subsequently aligned to the human genome using Bowtie2 (v2.3.4.1). Aligned reads were filtered using Samtools (v1.9) and duplicates were removed with Picard. Insertion sites were offset-corrected using GenomicRanges (v1.34.0) in R and normalized coverage bigwig files were constructed using rtracklayer (v1.42.2) in R. Peak calling was performed using MACS2 (v2.2.6). The differential accessibility analysis was performed with edgeR (v3.24.3). Annotation of peaks was performed using ChIPseeker (v1.18.0).

TT-seq
Raw reads were filtered using fastp (v0.13.1) and subsequently aligned to the human genome and the yeast genome using STAR (v2.7.9a), respectively. SAMtools (v0.1.19) and Picard were used to sort, index and mark duplicate reads in the resulting genome BAM files. Scale factor for each sample were estimated, and the differential expression analysis was performed with DESeq2 (v1.22.2). Strand-specific metagene profiles were generated with Ngs.plot.

RNA-seq
Raw reads were filtered using fastp (v0.13.1) and subsequently aligned to the human genome and the mouse genome using HISAT2 (v2.1.0). Gene level read count were calculated using featureCounts (v1.6.1). The differential expression analysis was performed with DESeq2 (v1.22.2). KEGG pathway enrichment analyses were performed with clusterProfiler (v3.10.1).

Read count quantification
Reproducibility of two biological replicates were assessed using Pearson correlation coefficient calculated by deepTools (v3.3.1). BamCoverage from deepTools was used to generate bigwig files of normalized read coverage per 50-bp bin for CUT&Tag, ChIP-seq, 4sUDRB-seq and TT-seq. Metagene profile plots were generated using computeMatrix and plotProfile from deepTools unless otherwise stated.

For manuscripts utilizing custom algorithms or software that are central to the research but not yet described in published literature, software must be made available to editors and reviewers. We strongly encourage code deposition in a community repository (e.g. GitHub). See the Nature Portfolio guidelines for submitting code & software for further information.

Data

Policy information about availability of data

All manuscripts must include a data availability statement. This statement should provide the following information, where applicable:
- Accession codes, unique identifiers, or web links for publicly available datasets
- A description of any restrictions on data availability
- For clinical datasets or third party data, please ensure that the statement adheres to our policy

Next generation sequencing data have been submitted to GEO repository under accession number GSE164277 (the secure token: izkligwozdaflst).

Field-specific reporting

Please select the one below that is the best fit for your research. If you are not sure, read the appropriate sections before making your selection.

☒ Life sciences ☐ Behavioural & social sciences ☐ Ecological, evolutionary & environmental sciences

For a reference copy of the document with all sections, see nature.com/documents/nr-reporting-summary-flat.pdf

Life sciences study design

All studies must disclose on these points even when the disclosure is negative.

Sample size	No statistical methods were used to predetermine sample size.
Data exclusions	No used in this study.
Replication	Two biological replicates were generated initially for each genomic analysis. Two more biological replicates were added if discrepancies were found between the original two biological replicates.
Randomization	Experiments were not randomized.
Blinding	Not used in this study.

Reporting for specific materials, systems and methods

We require information from authors about some types of materials, experimental systems and methods used in many studies. Here, indicate whether each material, system or method listed is relevant to your study. If you are not sure if a list item applies to your research, read the appropriate section before selecting a response.

Materials & experimental systems

n/a	Involved in the study	
☐	☒	Antibodies
☐	☒	Eukaryotic cell lines
☒	☐	Palaeontology and archaeology
☒	☐	Animals and other organisms
☒	☐	Human research participants
☒	☐	Clinical data
☒	☐	Dual use research of concern

Methods

n/a	Involved in the study	
☐	☒	ChIP-seq
☒	☐	Flow cytometry
☒	☐	MRI-based neuroimaging

Antibodies

Antibodies used	Pol II, Santa Cruz, sc-899; Pol II (Ser-2p), clone 3E10, Active Motif, 61083, lot 32418002; Pol II (Ser-5p), clone 3E8, Active Motif, 61085, lot 10618002; TOX4, Bethyl, A304-873A, lot 1; PPP1CC, Bethyl, A300-906A, lot 1; NELF-E, Proteintech, 10705-1-AP, lot 00023389; SPT5, clone D-3, Santa Cruz, sc-133217, lot J1416; SPT5, Bethyl, A300-868A, lot 1; p-SPT5 Thr806, Fisher Lab; PNUTS, Bethyl, A300-439A, lot 3; WDR82, Cell Signaling, 99715S; TBP, clone 1TBP18, Santa Cruz, sc-56795, lot L0214; PP1α, Santa Cruz, sc-271762, lot E2319; PP1β, Santa Cruz, sc-373782, lot J1817; PP1α, Bethyl, A300-904A, lot 2; PP1β, Bethyl, A300-905A, lot 1.
Validation	All the antibodies were validated by either the vendor or the contributor.<br>Pol II: https://www.citeab.com/antibodies/825104-sc-899-pol-ii-n-20<br>Pol II (Ser-2p): https://www.activemotif.com.cn/catalog/details/61083/rna-pol-ii-ctd-phospho-ser2-antibody-mab<br>Pol II (Ser-5p): https://www.activemotif.com.cn/catalog/details/61085/rna-pol-ii-ctd-phospho-ser5-antibody-mab<br>TOX4: https://www.bethyl.com/product/A304-873A/TOX4+Antibody<br>PPP1CC: https://www.bethyl.com/product/A300-906A/PPP1CC+Antibody<br>NELF-E: https://www.ptgcn.com/products/RDBP-Antibody-10705-1-AP.htm<br>SPT5(Santa Cruz): https://www.scbt.com/p/spt5-antibody-d-3?requestFrom=search<br>SPT5(Bethyl): https://www.bethyl.com/product/A300-868A/SUPT5H+Antibody<br>p-SPT5 Thr806: https://doi.org/10.1038/s41467-020-18173-6<br>PNUTS: https://www.bethyl.com/product/A300-439A/PNUTS+Antibody<br>WDR82: https://www.cellsignal.cn/products/primary-antibodies/wdr82-d2i3b-rabbit-mab/99715?_=1628584295677&Ntt=WDR82&tahead=true<br>TBP: https://www.scbt.com/p/tfiid-antibody-1tbp18?requestFrom=search<br>PP1α(Santa Cruz): https://www.scbt.com/p/pp1alpha-antibody-g-4<br>PP1β(Santa Cruz): https://www.scbt.com/p/pp1beta-antibody-c-5<br>PP1α(Bethyl): https://www.bethyl.com/antibody/pca_a-z/PPP1CA<br>PP1β(Bethyl): https://www.bethyl.com/product/A300-905A/PPP1CB+Antibody

Eukaryotic cell lines

Policy information about cell lines

Cell line source(s)	K562, 293T, High Five and SF9 cell line from ATCC
Authentication	The cell line were not authenticated.
Mycoplasma contamination	The cells were found free of mycoplasma contamination by PCR.
Commonly misidentified lines<br>(See ICLAC register)	No commonly misidentified cell lines were used.

ChIP-seq

Data deposition

☒ Confirm that both raw and final processed data have been deposited in a public database such as GEO.

☒ Confirm that you have deposited or provided access to graph files (e.g. BED files) for the called peaks.

Data access links<br>*May remain private before publication.*	https://www.ncbi.nlm.nih.gov/geo/query/acc.cgi?acc=GSE164277
Files in database submission	Raw data in fastq format, coverage data in bigwig files are deposited to GEO.
Genome browser session<br>(e.g. UCSC)	http://genome.ucsc.edu/s/aiwei/hg38_TOX4_ChIP%2Dseq

Methodology

Replicates	Two biological replicates
Sequencing depth	All ChIP-seq data were sequenced with pair-end 150bp reads.<br><br>Control TOX4 ChIP-seq replicate1:<br>35015164 reads; of these:<br>35015164 (100.00%) were paired; of these:<br>1905625 (5.44%) aligned concordantly 0 times<br>28984148 (82.78%) aligned concordantly exactly 1 time<br>4125391 (11.78%) aligned concordantly >1 times<br>----<br>1905625 pairs aligned concordantly 0 times; of these:<br>1155155 (60.62%) aligned discordantly 1 time<br>----

750470 pairs aligned 0 times concordantly or discordantly; of these:
1500940 mates make up the pairs; of these:
776515 (51.74%) aligned 0 times
319725 (21.30%) aligned exactly 1 time
404700 (26.96%) aligned >1 times
98.89% overall alignment rate

Control TOX4 ChIP-seq replicate2:
30781761 reads; of these:
30781761 (100.00%) were paired; of these:
2791161 (9.07%) aligned concordantly 0 times
24147179 (78.45%) aligned concordantly exactly 1 time
3843421 (12.49%) aligned concordantly >1 times

2791161 pairs aligned concordantly 0 times; of these:
849453 (30.43%) aligned discordantly 1 time

1941708 pairs aligned 0 times concordantly or discordantly; of these:
3883416 mates make up the pairs; of these:
3332650 (85.82%) aligned 0 times
222848 (5.74%) aligned exactly 1 time
327918 (8.44%) aligned >1 times
94.59% overall alignment rate

Antibodies

TOX4 antibody from Bethyl (A304-873A)

Peak calling parameters

Peak calling was performed using MACS2 (v2.2.6) with following parameters: -g hs -q 0.000001 --nomodel

Data quality

21711 and 27281 significant peaks (q<0.000001) were identified in each replicate, respectively.

Software

Bowtie2 (v2.3.4.1) for mapping; MACS2 (v2.2.6) for peak calling.
